# The chicken gut metagenome and the modulatory effects of plant-derived benzylisoquinoline alkaloids

**DOI:** 10.1186/s40168-018-0590-5

**Published:** 2018-11-27

**Authors:** Peng Huang, Yan Zhang, Kangpeng Xiao, Fan Jiang, Hengchao Wang, Dazhi Tang, Dan Liu, Bo Liu, Yisong Liu, Xi He, Hua Liu, Xiubin Liu, Zhixing Qing, Conghui Liu, Jialu Huang, Yuwei Ren, Long Yun, Lijuan Yin, Qian Lin, Cheng Zeng, Xiaogang Su, Jingyang Yuan, Li Lin, Nanxi Hu, Hualiang Cao, Sanwen Huang, Yuming Guo, Wei Fan, Jianguo Zeng

**Affiliations:** 1 Hunan Key Laboratory of Traditional Chinese Veterinary Medicine, Hunan Agricultural University, Changsha, 410128 Hunan China; 2 College of Horticulture and Landscape, Hunan Agricultural University, Changsha, 410128 Hunan China; 3 Agricultural Genomic Institute, Chinese Academy of Agricultural Sciences, Shenzhen, 518120 Guangdong China; 4 State Key Laboratory of Animal Nutrition, College of Animal Science and Technology, China Agricultural University, Beijing, 100193 China; 5 College of Veterinary Medicine, Hunan Agricultural University, Changsha, 410128 Hunan China; 6 College of Animal Science and Technology, Hunan Agricultural University, Changsha, 410128 Hunan China; 7 National and Local Union Engineering Research Center of Veterinary Herbal Medicine Resource and Initiative, Hunan Agricultural University, Changsha, 410128 Hunan China

**Keywords:** Chicken, Gut metagenome, Microbiome, Growth promoter, Benzylisoquinoline alkaloid, Antibiotic, Chlortetracycline

## Abstract

**Background:**

Sub-therapeutic antibiotics are widely used as growth promoters in the poultry industry; however, the resulting antibiotic resistance threatens public health. A plant-derived growth promoter, *Macleaya cordata* extract (MCE), with effective ingredients of benzylisoquinoline alkaloids, is a potential alternative to antibiotic growth promoters. Altered intestinal microbiota play important roles in growth promotion, but the underlying mechanism remains unknown.

**Results:**

We generated 1.64 terabases of metagenomic data from 495 chicken intestinal digesta samples and constructed a comprehensive chicken gut microbial gene catalog (9.04 million genes), which is also the first gene catalog of an animal’s gut microbiome that covers all intestinal compartments. Then, we identified the distinctive characteristics and temporal changes in the foregut and hindgut microbiota. Next, we assessed the impact of MCE on chickens and gut microbiota. Chickens fed with MCE had improved growth performance, and major microbial changes were confined to the foregut, with the predominant role of *Lactobacillus* being enhanced, and the amino acids, vitamins, and secondary bile acids biosynthesis pathways being upregulated, but lacked the accumulation of antibiotic-resistance genes. In comparison, treatment with chlortetracycline similarly enriched some biosynthesis pathways of nutrients in the foregut microbiota, but elicited an increase in antibiotic-producing bacteria and antibiotic-resistance genes.

**Conclusion:**

The reference gene catalog of the chicken gut microbiome is an important supplement to animal gut metagenomes. Metagenomic analysis provides insights into the growth-promoting mechanism of MCE, and underscored the importance of utilizing safe and effective growth promoters.

**Electronic supplementary material:**

The online version of this article (10.1186/s40168-018-0590-5) contains supplementary material, which is available to authorized users.

## Background

Global chicken production makes a substantial contribution to food security. Although sub-therapeutic antibiotics have been widely used as growth promoters (AGPs) in livestock to maintain health and enhance productivity, the resulting antibiotic resistance has become a major threat to public health [1]. The European Union has banned the use of AGPs since 2006 [2]; thus, the development of safe alternatives to AGPs has become a global focus.

The growth-promoting mechanisms of AGPs are only partially understood. Germ-free chickens do not gain weight in response to low-dose antibiotic [3]. It is the altered gut microbiota that plays a causal role, not antibiotics, per se [4]. The effects of AGPs are generally thought to be through inhibition of sub-clinical infections, reduction of growth-depressing metabolites from gut microbiota, reduction of nutrients available for pathogens, and so forth [5]. However, it is still unclear how sub-therapeutic antibiotics could efficiently prevent infection and promote growth, and hence, further studies are needed to advance our understanding of AGPs.

Natural growth promoters (NGPs), such as probiotics, prebiotics, and phytobiotics, have been exploited, as alternatives to antibiotics in livestock production. In fact, most NGPs take effect through altering the gut microbiota. Probiotics are living microorganisms that confer benefits to the host, examples being *Lactobacillus* and *Bifidobacterium* [6]. Prebiotics are substrates selectively utilized by gut microbiota and include non-digestible oligosaccharides and polyunsaturated fatty acids [7]. Phytobiotics represent a wide range of plant-derived bioactive compounds, which confer multiple effects to the host, and can also stimulate beneficial bacteria in the gut [8].

The phytobiotic *Macleaya cordata* extract (MCE) has been widely used, for decades, in feed livestock in many countries [9]. The effective chemical composition of MCE includes sanguinarine and chelerythrine, both belonging to a group of benzylisoquinoline alkaloids, which have antimicrobial and anti-inflammatory properties [10–12]. Additionally, sanguinarine has a molecular structure highly similar to another benzylisoquinoline alkaloid, berberine, which is clinically effective in treating some diseases by modulation of the gut microbiota [13, 14]. Nevertheless, details concerning the mechanism(s) associated with growth promotion remain unclear.

Currently, the gut microbial gene catalogs of humans, mice, and pigs have been established [15–18], which greatly facilitated gut microbial studies in the health and diseases in these hosts. In this study, we constructed the first comprehensive chicken gut microbial gene catalog, to better understand the related microbiota. We then systematically studied the impact of MCE and the commonly used AGP, chlortetracycline (CTC). The gut metagenome analysis provided a deeper insight into the growth promoters.

## Results and discussion

### A comprehensive chicken gut microbial gene catalog and its comparison with the human and pig catalogs

**Fig. 1:**
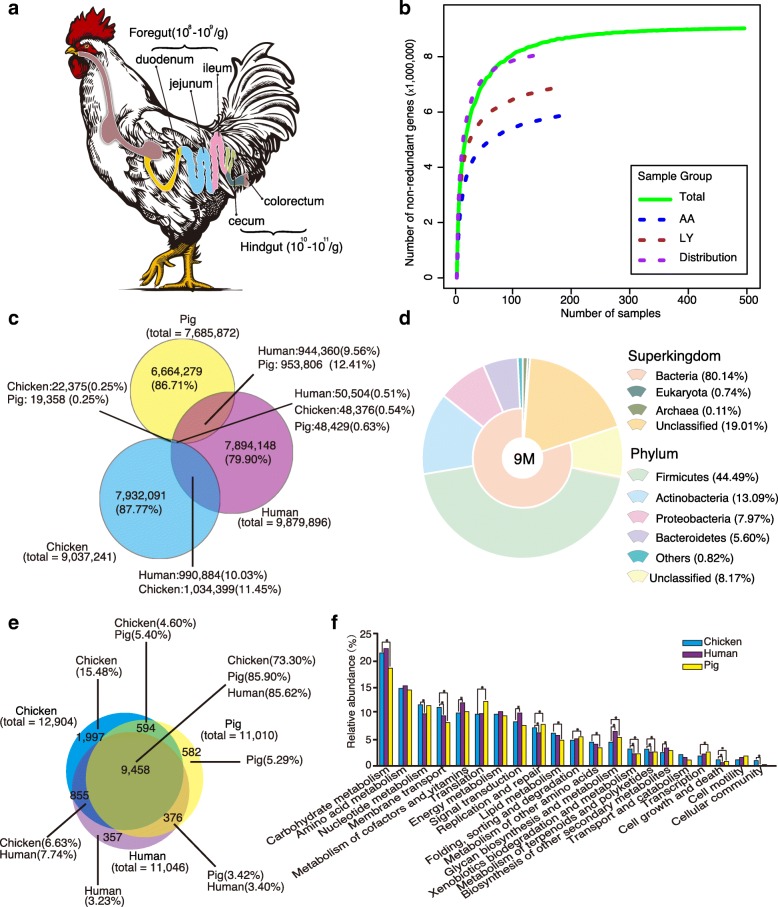
Chicken gut microbial gene catalog. **a** Diagram of chicken intestinal tract. The microbial densities in the foregut and hindgut were labeled. **b** Rarefaction curves of detected genes from the whole set of 495 samples (Total) and from subgroups of LY, AA, and Distribution. A total of 9.04 million non-redundant genes were detected, and the rarefaction curve including all samples approaches saturation at the end of sampling. The gene number of a specific number of samples was calculated after random samplings repeated 100 times with replacement, and the median was plotted. **c** Venn diagram of gut microbial genes shared between the chicken, human, and pig catalogs. The criteria for shared genes were sequence identity > 95% and overlap > 90% of the shorter gene. **d** Taxonomic annotation of the chicken gut gene catalog at the superkingdom and phylum levels. **e** Venn diagram of KEGG orthologous groups (KOs) present in and shared by chicken, human, and pig catalogs. **f** Comparison of KEGG functional profiles (relative gene abundance summarized into KEGG functional categories and genes without functional annotations were excluded) of gut microbiome among chickens, humans, and pigs. Asterisks denote Wilcoxon rank-sum test result (*P* < 0.005)

We collected 495 digesta samples from the five intestinal compartments (duodenum, jejunum, ileum, cecum, and colorectum) of chickens raised in 7 different farms in China. These chickens varied in breeds (7 breeds: Hy-Line Variety Brown, Cobb 500, Ross 308, Arbor Acres broiler, Local yellow-feather chickens, Yellow dwarf chicken, and Guangxi local chicken), farming systems (battery-cage and free-range), and farm location (6 Chinese provinces) (Fig. 1a and Additional file 1: Table S1). High-throughput sequencing generated a total of 1.64 terabases (Tb) of clean metagenomic data, without low-quality reads, adapter or host contaminants, with an average of 3.31 gigabases (Gb) per sample. Based on the assembled contigs with an N50 contig length of 1.95 kb, we identified 9.04 million non-redundant genes, with an average open reading frame (ORF) length of 697 bp.

Rarefaction analysis of all samples revealed a curve approaching saturation (Fig. 1b, Additional file 2: Figure S1), suggesting that the vast majority of chicken gut microbial genes are present in our gene catalog. In fact, the size and quality of this chicken gene catalog are comparable to those of human (9.9 million genes) and pig (7.7 million genes) catalogs (Additional file 3: Table S2), which provided useful reference genes for subsequent studies [16, 17]. By comparing the pairwise overlap, at the gene sequence level, we determined that over 80% of genes are unique to each species, and only a very small percentage (~ 0.5%) of genes are shared by chickens, humans, and pigs. Interestingly, chickens and pigs share fewer microbial genes (~ 0.8%) than do chickens and humans (~ 10%) or pigs and humans (~ 10%) (Fig. 1c), the latter of which is consistent with a previous report [17].

**Fig. 2:**
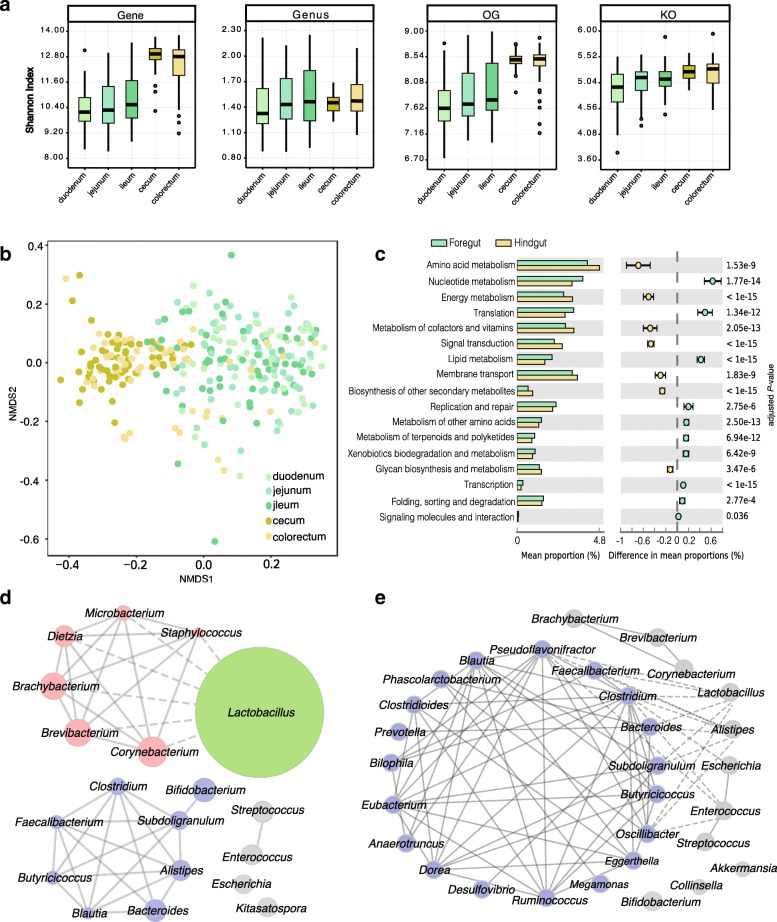
Comparison of gut microbiome in different intestinal compartments of chickens. **a** Microbial diversity (Shannon index) at gene, genus, OG, and KO levels. Box plots show median ± interquartile range (IQR) and 1.5 IQR ranges (whiskers), with outliers denoted by dots. **b** The non-metric multidimensional scaling (NMDS) plot based on Bray-Curtis dissimilarities at species level. An obvious difference was observed between the foregut (duodenum, jejunum, and ileum) and hindgut (cecum and colorectum). **c** Differences in microbial functions between the foregut and hindgut based on KEGG functional categories (Wilcoxon rank-sum test, Storey’s methods for multiple tests adjustment). Chicken gut microbial co-occurrence network analysis based on core genus (average relative abundance > 0.1%) **d** in the foregut and **e** hindgut. Solid line: Spearman’s rank correlation coefficient > 0.30; dash line: Spearman’s rank correlation coefficient < − 0.30. The size of nodes was proportional to the relative abundance of genera

Using CAMRA3 for taxonomic assignment, 80.99% of the genes in the chicken gut catalog were taxonomically classified at the superkingdom level, among which bacteria account for 98.95% of the classified genes, with the remaining 1% being from archaea and eukaryotes. More than 88% of the bacterial genes are from the top four phyla, including *Firmicutes*, *Actinobacteria*, *Proteobacteria*, and *Bacteroidetes* (Fig. 1d). In the human and pig gut catalogs, *Firmicutes* and *Bacteroidetes* are predominant, and *Proteobacteria* and *Actinobacteria* make up a smaller percentage [16, 17]. At lower taxonomic levels, 25.97% and 2.29% of the genes in this catalog were annotated to genus and species, respectively. Note that the short-chain fatty acid (SCFA)-producing genera, such as *Bacteroides*, *Blautia*, *Ruminococcus*, and *Faecalibacterium*, are among the major genera in both human and pig guts. Similarly, in the chicken gut, these genera are also among the major genera of relatively high abundance (Fig. 2d, Additional file 4: Table S3), indicating the importance of these gut microbes in both birds and mammals.

Using KEGG and eggNOG for function gene classification, 5,454,369 (60.4%) and 6,881,483 (76.1%) genes in the catalog were annotated with KEGG orthologous groups (KOs) and eggNOG orthologous groups (OGs), respectively; values are comparable to those of human and pig catalogs (Additional file 5: Figure S2). As shown by Venn diagrams (Fig. 1e and Additional file 6: Figure S3), a large majority of the KOs (73–86%) and OGs (46–77%) were shared among chickens, humans, and pigs, representing shared gut microbial functions, despite vast differences at the gene sequence level.

The KEGG functional profiles, based on the functional assignments and relative gene abundances, also showed similarities in gut microbial functions in the different hosts (Fig. 1f). However, there were still significant differences (*P* < 0.005) in some KEGG functional categories, and the non-metric multidimensional scaling (NMDS) analysis of KOs also showed clear differences among chicken, human, and pig gut samples (Additional file 7: Figure S4). Notably, the genes for glycan biosynthesis and metabolism were more abundant in human and pig guts, whereas the genes for membrane transport, an essential mechanism for the uptake of substrates, such as sugars, lipids, peptides, and ions, were more abundant in the chicken gut microbiota (Fig. 1f). The enriched genes for membrane transport likely reflect the availability of more nutrient substrates, in the chicken intestine, for direct microbial utilization. The more abundant genes for metabolism of xenobiotics, terpenoids, and polyketides, in the chicken gut, are relevant to the abundant bacterial phylum *Actinobacteria*, which decomposes organic matter and produces various natural drugs, enzymes, and bioactive metabolites [19].

With the first comprehensive chicken gut catalog and the diverse samples from different farms (DHC, DGY, DHK, DSL, and DST) (Additional file 1: Table S1), we determined that the taxonomic variability among samples was high (Additional file 8: Figure S5), and the NMDS plot showed separations among chicken groups from different farms (Additional file 9: Figure S6). Notably, the intestinal microbial diversities (Shannon index) of chickens in free-range farming (DHC and DGY) were higher than those in battery-cage (DHK, DSL and DST) systems (Additional file 10: Figure S7). In addition, *Actinobacteria*, which is a dominant soil phylum, was more abundant in free-range farming than in battery-cage chickens (Additional file 11: Figure S8). The observed differences were associated with lifestyles, as free-range chickens were exposed to the outdoor environment and came into contact with more diverse microbes that shape their different gut microbiota.

### Distinctive characteristics of chicken foregut and hindgut metagenomes

Distinguished by the difference in morphology and function, the chicken intestinal tract can be divided into the foregut and hindgut. The foregut contains the duodenum, jejunum, and ileum compartments. The duodenum has the major function in feed digestion by using digestive enzymes and bile from the pancreas and liver, and the released nutrients, such as amino acids, fatty acids, sugars, and peptides, are mainly absorbed in the jejunum and ileum. The hindgut contains the cecum and colorectum. Substantial microbial fermentation occurs in the cecum, which provides nutrients, detoxifies some harmful substances, and also helps to prevent pathogen colonization [20, 21]. The colorectum is the distal part of the intestinal tract, where residual water and salt absorption occurs [22]. Previous chicken gut studies focused more on cecal or fecal microbiota [23–25], and some microbial functions such as polysaccharide utilization, SCFAs production, and hydrogen consumption in the cecum have been studied [26, 27]. The microbiota in the foregut where nutrient absorption primarily occurs were mostly studied by 16S rRNA gene sequencing with limited sample size [28, 29]. In this part of our study, metagenomic data of 285 samples from all five intestinal compartments in chickens older than 40 days were analyzed (Fig. 1a and Additional file 1: Table S1).

Based on the relative abundance of genes, genus, OGs, and KOs, we examined the microbial diversities (Shannon indexes) in each intestinal compartment (Fig. 2a). Our results indicated that microbial diversities in the foregut compartments were approximately the same, with only a slight increase from duodenum, jejunum, to ileum. A similar situation was observed between the hindgut cecum and colorectum; however, the diversities were clearly higher in the hindgut than in the foregut compartment (Fig. 2a).

We next calculated the number of common genes presented in 50% of the samples from each intestinal compartment, and the results showed that the common genes accounted for only about 2% of all genes in each foregut compartment (duodenum, jejunum, and ileum), but the number increased to 18% and 13% in the cecum and colorectum, respectively (Additional file 12: Table S4). The NMDS analysis revealed that there was a clear separation between the foregut and hindgut compartments (Fig. 2b). Taken together, these results indicated an overall similarity within foregut and hindgut compartments, with a larger difference between them, which is consistent with previous studies [29, 30].

The relative abundance profiles of phylum and genus showed distinct microbial features between the foregut and hindgut samples (Additional file 8: Figure S5). At the phylum level, *Actinomycetes* and *Bacteroides* showed significant differences (*P* < 0.001) and were twofold enriched in the foregut and sixfold enriched in the hindgut, respectively (Additional file 4: Table S3). As previously reported [29, 31], at the genus level, *Lactobacillus* is the predominant genus in the foregut, but not in the hindgut (Additional file 4: Table S3); *Lactobacillus* provides nutrients to the host and defends against opportunistic pathogens [32, 33]. Moreover, the relative abundance of genera, such as *Corynebacterium*, *Brevibacterium*, and *Brachybacterium*, in the foregut was higher than that in the hindgut (Additional file 4: Table S3). By contrast, a variety of anaerobic genera, such as *Subdoligranulum*, *Bacteroides*, *Faecalibacterium*, *Clostridium*, and *Butyricicoccus*, were more abundant in the hindgut (Additional file 4: Table S3).

By using the 285 samples from chickens older than 40 days, we constructed the co-occurrence network of the core genera in both foregut and hindgut. In the foregut, *Lactobacillus* competitively inhibits a cluster of bacteria, with negative correlations with all these genera (Fig. 2d). Additionally, some SCFA producers, such as *Clostridium*, *Butyricicoccus*, and *Faecalibacterium*, showed positive correlations with one another and form a relatively independent and stable cluster (Fig. 2d). In the hindgut, 19 genera are positively correlated with each other and form a large co-occurrence network (Fig. 2e). Some of these bacteria are beneficial intestinal microbes that produce metabolites, such as SCFAs, by fermentation [26, 34], while opportunistic pathogenic bacteria, *Escherichia* and *Enterococcus*, are inhibited by the central microbial cluster (Fig. 2e). These results revealed more diverse and complex microbial communities in the hindgut than in the foregut.

According to our KEGG functional analysis (Fig. 2c and Additional file 13: Figure S9), the microbiota in the foregut was enriched in genetic information processing for transcription, translation, and replication, as well as the metabolic functions of nucleotides and lipids, whereas microbes in the hindgut were enriched for the metabolic functions of amino acids, energy metabolism, and secondary metabolite biosynthesis. Similar results were obtained based on eggNOG analysis (Additional files 14 and 15: Figure S10 and S11). These findings were consistent with the substantial microbial fermentation in the hindgut, and the production of various metabolites, such as amino acids and SCFAs that are important for host health [35–37]. By taking the taxonomic features into consideration, we noticed that *Lactobacillus* has a relatively small genome (2 Mb) compared to other bacteria, and encodes higher proportion of genes for basic functions such as transcription, translation, and replication, but lower proportion of genes for many diverse metabolic functions. Therefore, the predominance of *Lactobacillus* in the foregut has largely contributed to the functional differences between the foregut and hindgut microbiome. In conclusion, the taxonomic and functional features of the foregut and hindgut microbiome are consistent with the morphological and physiological structure of the chicken intestine.

### Temporal development and maturation of the chicken gut microbiome

**Fig. 3:**
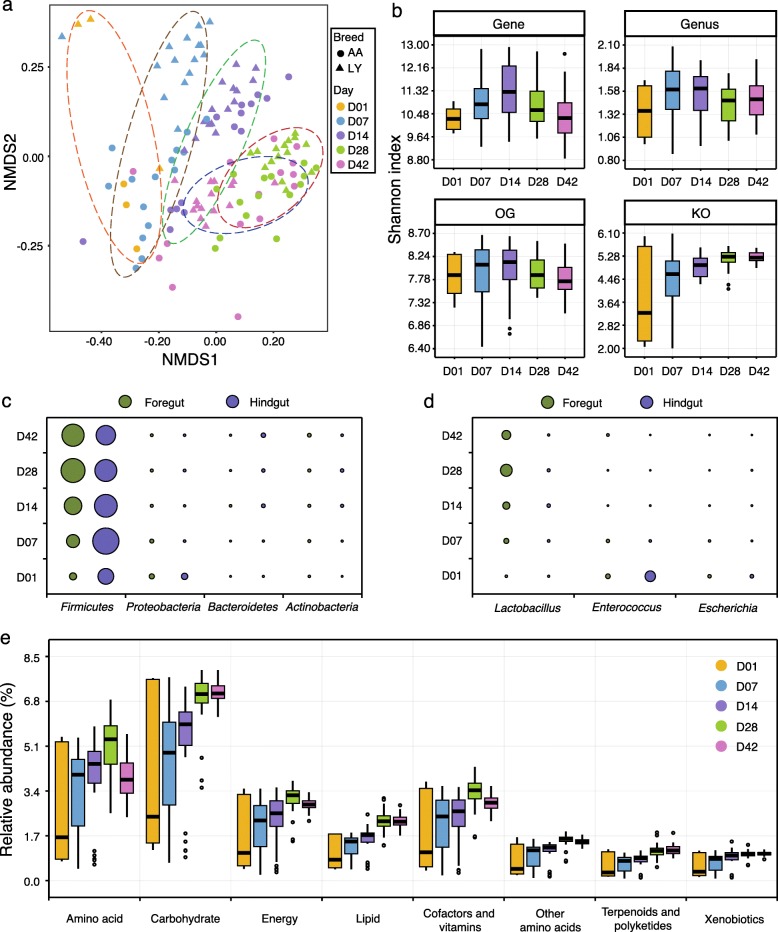
Differences in the chicken intestinal microbiome at different ages. **a** The NMDS plot of microbial communities in the foregut at different ages. The analysis was based on Bray-Curtis dissimilarities at the species level, and samples were grouped according to the ages. **b** Microbial diversity (Shannon index) in the foregut at gene, genus, OG, and KO levels. Box plots show median ± interquartile range (IQR) and 1.5 IQR ranges (whiskers), with outliers denoted by dots. The relative abundance changes in major **c** phyla and **d** genera at different ages in both the foregut and hindgut. The area of the circles represents the relative abundance of each phylum and genus. **e** Relative abundance of KEGG metabolic pathways of the microbiome in the foregut at different ages

To investigate the development of gut microbial communities, samples of five different chicken ages (1, 7, 14, 28, and 42 days) were analyzed for two breeds, Arbor Acres (AA) and Local yellow-feather (LY). Previously, researchers have found that the microbiota were inherited partly from maternal hens, and then influenced by environmental factors [38]. Our data showed that the gut microbiome of newly hatched chicks (day 1) were highly variable and obviously different from other samples (day 7, 14, 28, and 42) (Fig. 3), reflecting the short-term exposure to the environment and the initiation of gut microbial communities.

The microbial development is influenced by many factors, such as diet, feed additive, and host breed, and the successional changes have been reported for a few chicken breeds [39–41]. In our study, for both AA and LY chickens, the NMDS plots showed that samples were clustered into groups by ages, with day 28 and 42 groups exhibiting much higher similarity (Fig. 3a and Additional file 16: Figure S12), which also revealed a successional development. Microbial diversities increased during chicken development, peaking at approx. day 14 and 28 for the foregut and hindgut, respectively, and then remaining stable or decreasing slightly thereafter (Fig. 3b and Additional file 17: Figure S13). Both the NMDS and microbial diversity analyses indicated that the intestinal microbiota develops into a relatively mature community, as the host chicken matures, and then, a stable state is maintained.

*Firmicutes*, *Proteobacteria*, *Bacteroidetes*, and *Actinobacteria* were the dominant phyla in both foregut and hindgut throughout the growth test, and they exhibited obvious temporal changes (Fig. 3c). The most abundant phylum, *Firmicutes*, increased in the foregut from day 1 to day 28, and then remained relatively stable until day 42, whereas in the hindgut, it slowly decreased from day 7 to day 42 (Fig. 3c and Additional file 18: Table S5). The predominant genus in the foregut, *Lactobacillus* (phylum: *Firmicutes*), changed in a similar way to *Firmicutes* (Fig. 3d and Additional file 19: Table S6). In the hindgut, major genera, such as *Lactobacillus*, *Subdoligranulum*, and *Bifidobacterium*, were more abundant in the middle growth period, while other genera, such as *Alistipes*, were more abundant at the end of the growth test (Additional file 20: Figure S14). In addition, the frequently reported zoonotic pathogens such as *Salmonella* and *Campylobacter* could be detected in both foregut and hindgut throughout the growth test, but for the reason that only healthy chickens were studied, both of the pathogens were detected at very low levels (average relative abundance, 0.01–0.03%) and their impacts might be much weaker than those in the infected chickens [42, 43]. In both foregut and hindgut, the metabolic capacity reached a maximum at day 28, thereafter remaining stable. However, differences among the ages were larger in the foregut than those in the hindgut (Fig. 3e and Additional file 21: Figure S15). The findings showed that the early days are critical both for chicken development and establishment of the gut microbiota.

### CTC and MCE promote chicken growth

To examine the effects of MCEs, compared with antibiotics, we next performed parallel experiments with two chicken breeds, each with five test groups that received CTC supplementation and three MCE gradient dosages (MCE-L, MCE-M, MCE-H), as feed additives, as well as a blank control (BLANK). The LY and AA chicken breeds were independently raised on two farms and measurements taken at 56 and 42 days, respectively.

In LY chickens (day 56), dietary supplements numerically (not significantly) improved the average body weight gain by 3.1% (CTC, *P* = 0.258), 2.2% (MCE-L, *P* = 0.204), 4.8% (MCE-M, *P* = 0.071), and 3.1% (MCE-H, *P* = 0.069), and the feed intakes were also significantly (*P* < 0.05) increased by 3.0–6.9% (Table 1). Therefore, the feed conversion ratio (FCR) was not significantly changed (*P* > 0.05). In AA chickens (day 42), the average body weight gain was also numerically (not significantly) improved by 1.5% (CTC, *P* = 0.363), 2.2% (MCE-M, *P* = 0.258), and 1.7% (MCE-H, *P* = 0.302) (Table 1). However, the increase of feed intake in AA chickens was not significant (*P* > 0.05). As a result, the FCR was significantly decreased (*P* < 0.05, from 1.77 to 1.71 in MCE-M), indicating the improvement of the nutrient absorption, which benefits the chicken farming. These results are consistent with previous findings that CTC and MCE could promote the body weight gain (2–5%), by improving either food intake or feed conversion efficiency [44–47]. However, the effects of MCE and CTC appeared more pronounced for large-scale chicken farms, probably due to the differences in farming conditions. In addition, chicken breeds, diets, and other factors also influence the effects of CTC and MCE [44–46], suggesting the complex mechanisms on growth promotion.

**Table 1:** Chicken growth performance in response to CTC and MCE treatments

Group	Local yellow-feather chickens (day 56)			Arbor Acre chickens (day 42)		
	Feed intake (g)	Body gain (g)	Feed conversion ratio	Feed intake (g)	Body gain (g)	Feed conversion ratio
BLANK	3443.13 ± 37.87^b^	1505.37 ± 49.46	2.33 ± 0.05	4462.13 ± 73.48	2519.13 ± 28.14	1.78 ± 0.02^a^
CTC	3547.64 ± 63.51^a^	1551.70 ± 39.45	2.31 ± 0.05	4522.67 ± 80.36	2557.41 ± 40.17	1.77 ± 0.01^a^
MCE-L	3597.37 ± 45.27^a^	1538.34 ± 42.02	2.32 ± 0.79	4437.84 ± 89.70	2510.00 ± 45.12	1.77 ± 0.01^a^
MCE-M	3681.41 ± 126.91^a^	1577.95 ± 83.28	2.37 ± 0.06	4405.79 ± 88.12	2575.28 ± 45.66	1.71 ± 0.01^b^
MCE-H	3627.05 ± 184.14^a^	1551.34 ± 27.37	2.31 ± 0.07	4489.43 ± 73.73	2562.08 ± 38.05	1.75 ± 0.01^a^
*P* value	0.018	0.163	0.361	0.869	0.728	0.004

Data are presented as mean ± SD; data in columns with no common superscript differ significantly (*P* < 0.05). Feed conversion ratio (feed intake/weight gain). Data for growth performance were analyzed with one-way ANOVA and Duncan’s multiple comparison in SPSS Version 18.0 (SPSS Inc., Chicago, Illinois, USA)

### CTC and MCE reshape the chicken foregut microbiota to promote growth

**Fig. 4:**
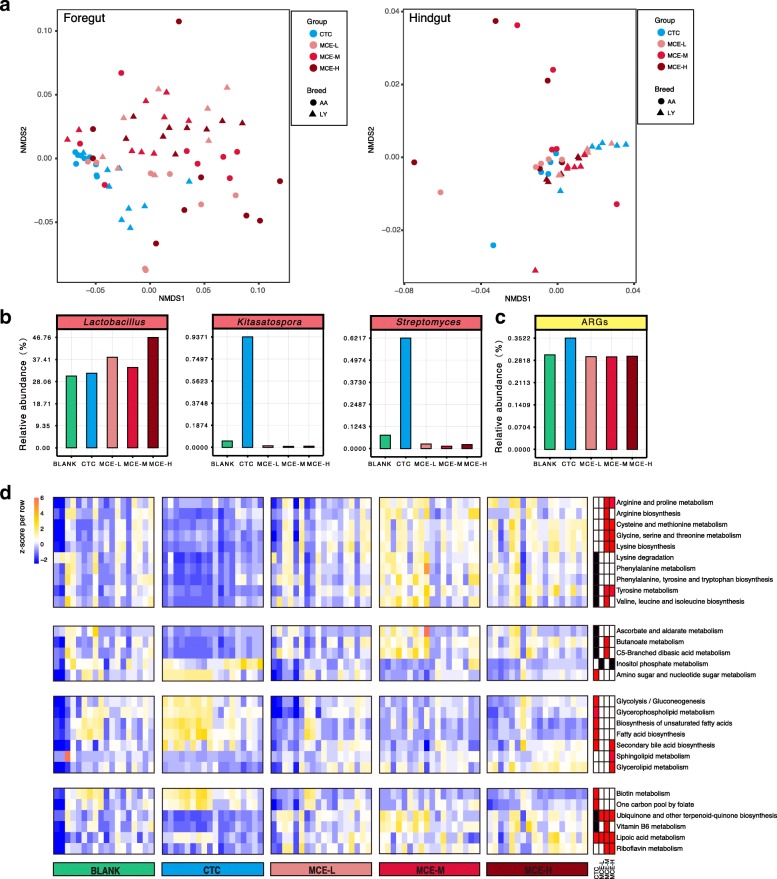
Differences in microbial changes after CTC and MCE treatment. **a** The NMDS plot of microbial communities in CTC and MCE groups, based on Bray-Curtis dissimilarities at the species level. The obvious difference was in the foregut. **b** The average relative abundances of genera increased by MCE or CTC in the foregut. *Kitasatospora* and *Streptomyces* were significantly (*P* < 0.05) increased by CTC. **c** The average relative abundance of ARGs was increased (*P* < 0.1) by CTC in the foregut. **d** The heatmap of KEGG metabolic pathways significantly altered by CTC or MCE in the foregut (18 samples for each group, including 9 samples from AA chickens and 9 samples from LY chickens). The relative abundance of each pathway was colored according to the row z-score ((value – row mean)/row standard deviation). Red, black, and white rectangles at the right side of the heatmap represent significant increase (*P* < 0.05), significant decrease (*P* < 0.05), and no significant change (*P* > 0.05) compared to the BLANK, respectively. The Kruskal-Wallis test (Storey’s methods for adjustment) was followed by a post-hoc Wilcoxon rank-sum test

Major microbial responses to growth promoters were observed in the foregut, but not in the hindgut (Fig. 4, Additional files 22, 23, 24, 25, 26, and 27: Figures S16–S21 and Additional file 28: Table S7). This is consistent with a previous finding that treatment with an AGP, avilamycin, more strongly impact microbiota composition in the ileum than in the cecum [48]. Considering that the hindgut has a much higher microbial cell density and microbial diversity (Figs. 1a and 2a) [49], as well as a more complex microbial network compared to the foregut (Fig. 2d, e), the microbiota in the hindgut is more stable and less affected by feed additives. In addition, the chicken ceca are a pair of blind-ended pouches, which provide a relatively closed microbial environment, and thus, are more likely to be resilient to interference. Our findings support the notion that microbial regulation, by growth promoters, in the foregut plays a more important role.

In the foregut, the predominant genus, *Lactobacillus*, was influenced by MCE. The average relative abundance was increased by 12–54% in the three groups (Fig. 4b and Additional file 24: Figure S18a), and thus, the predominant role of *Lactobacillus* in the foregut was further strengthened, particularly by MCE-H. *Lactobacillus* is recognized as a beneficial probiotic that produces vitamins and organic acids, and also competitively inhibits pathogens [33, 50, 51]. Moreover, through the “cross-feeding” mechanism, the lactate produced by *Lactobacillus* could be used by anaerobic bacteria to produce butyrate [52], which is an important energy source for intestinal cells and exerts anti-inflammatory activities [53]. In the CTC group, *Lactobacillus* was increased (*P* > 0.05) by 4%, but this increase was lower than that in the MCE groups, and there were some inconsistencies regarding the impact of CTC on *Lactobacillus* [54, 55]. By contrast, CTC significantly (*P* < 0.05) enriched the antibiotic-producing genera of *Kitasatospora* and *Streptomyces* (Fig. 4b and Additional file 24: Figure S18a), which are both from the family *Streptomycetaceae* and produce a variety of antibiotics [56, 57]. In particular, 80% of currently used antibiotics are sourced from *Streptomyces* [57]. Consistent with these findings, we observed the enrichment of several antibiotic biosynthesis pathways in the CTC group (Additional file 24: Figure S18b), including those for tetracycline, macrolides, type II polyketide, and clavulanic acid. The presumably enhanced production of natural antibiotics would amplify the antimicrobial and anti-inflammatory effects of the administered antibiotic, and therefore benefit the host*.* On the other hand, however, we established that the antibiotic resistance genes (ARGs) were also increased (*P* = 0.097) in the CTC group (Fig. 4c and Additional files 24 and 25: Figures S18c and S19). These findings provide a new perspective to understand the complex impact of sub-therapeutic antibiotic treatment.

The relative abundances of some core genera, such as *Corynebacterium*, *Brachybacterium*, and *Dietzia*, which were in the same microbial co-occurrence network in the foregut (Fig. 2d), were significantly decreased in both MCE and CTC groups (Additional file 26: Figure S20). By comparing the three MCE groups, we found that the high dose had stronger inhibiting effect, and more genera were significantly (*P* < 0.05) decreased in MCE-H than in MCE-M and MCE-L (Additional file 26: Figure S20). This effect might be associated with the competitive inhibition effect of *Lactobacillus* (Fig. 2d), and the higher relative abundance of *Lactobacillus* in MCE-H. Most of the inhibited genera by MCE (Additional file 26: Figure S20) are normal, with no clear benefit or harm to the host, whereas genera such as *Corynebacterium* and *Microbacterium* also include pathogenic species that may severely threaten animal health [58, 59]. The decrease of pathogens is likely to alleviate the host inflammation and immune responses, and indeed, our data showed that the host cytokines including IL-4, IFN-γ, and NF-κB were downregulated (Additional file 29: Table S8). However, the modulatory effects of MCE were possibly not associated with the frequently reported pathogens such as *Salmonella* or *Campylobacter* [42, 43, 60], as their relative abundances were low in samples and little change was found after the MCE treatment. In addition, many other bacteria at similar or lower abundance levels may be involved in the modulation as well, which made the detailed mechanism more complicated. Overall, the positive regulation of beneficial *Lactobacillus*, and the negative regulation of some commensal and pathogenic bacteria, constitutes the overall foregut microbial compositional changes after the MCE treatment.

MCE significantly (*P* < 0.05) enriched the amino acid biosynthesis and metabolism pathways in the foregut (Fig. 4d). Microbial-synthesized amino acids are an important nutrient supplement for the host, and these molecules are primarily absorbed in the foregut rather than in the hindgut [61]. Dietary supplementation of amino acids, such as lysine and arginine, improved the body weight and feed conversion efficiency, as well as enhanced the immunity [61, 62]. Accordingly, the metagenomic results suggest that the growth promotion may be achieved by the enhancement of microbial amino acid biosynthesis.

In lipid metabolism, the secondary bile acid biosynthesis pathway was enriched by both MCE and CTC. The host-secreted bile acids have antimicrobial activities that alter the gut microbial composition. Meanwhile, the microbial modification of bile acids can facilitate fat absorption, and is therefore involved in regulating host energy metabolism and immune system [63, 64]. Besides, the biosynthesis pathways of fatty acids and unsaturated fatty acids that are closely related to host lipid metabolism were also enriched in the CTC group. This result indicated that the lipid metabolism regulation is an important mechanism for growth promoters.

Moreover, both MCE and CTC influenced the carbohydrate and vitamin metabolism pathways (Fig. 4d). Two sugar-related metabolism pathways were enriched by CTC, while C5-branched dibasic acid and butanoate (butyrate) metabolism pathways were enriched by MCE (Fig. 4d). The SCFA butyrate is a metabolic energy source for intestinal cells, and it has anti-inflammatory effects and helps the host to maintain mucosal barrier integrity [65, 66]. Vitamins are essential micronutrients for biochemical reactions, and dietary supplement with vitamins enhances the chicken immune system [67, 68]. The gut microbiota also acts as an important vitamin supplier for the host [33]. In the present study, the biotin and vitamin-like lipoic acid pathways were enriched in the CTC group (Fig. 4d). Particularly, lipoid acid supplements can improve the growth performance and antioxidant capacity of the host [69]. In the MCE groups, a greater variety of pathways were enriched, including lipoic acid, vitamin B_6_, riboflavin, ubiquinone, and other terpenoid-quinone (including vitamin K_1_, K_2_, and E) pathways (Fig. 4d). In summary, vitamins, in addition to other microbial synthesized nutrients, were enhanced by MCE and consequently benefited the host.

## Conclusions

Given the importance of chicken production in agriculture and the remarkable contribution of intestinal microbiota to the host’s nutrition and health, the chicken gut microbiota has received growing attention worldwide. In the present study, we constructed the first comprehensive gene catalog of the chicken gut microbiome, by using the digesta samples of all intestinal compartments of chickens from diverse farms in China, and of chickens at different ages throughout the growing period of broilers. Importantly, the foregut microbiome was less studied in either humans or other animals. Our metagenomic results emphasized the similarity of the microbiota within the foregut and hindgut compartments, but exhibited distinctive taxonomic and functional differences between them as well. The intestinal microbiota develops into a relative mature community and reaches the maximum metabolic capacity during day 15–28. These findings make an important supplement to the animal gut metagenomes, especially for chickens.

**Fig. 5:**
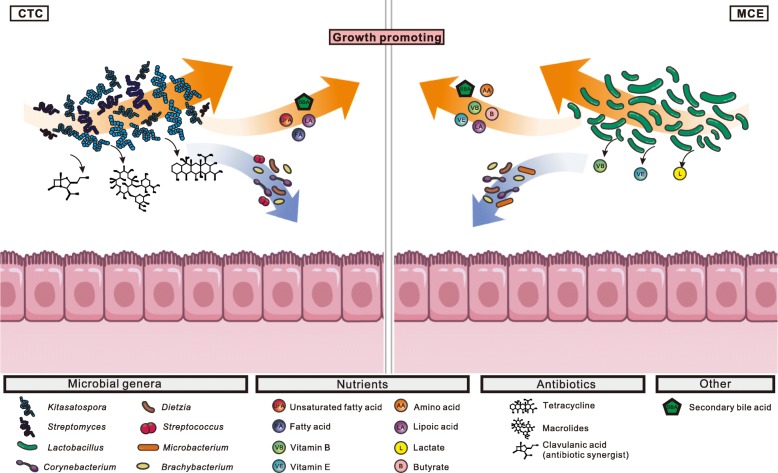
The putative mechanisms of growth promotion by altering the foregut microbiota through CTC and MCE treatment. (Left) The antibiotic CTC as an exogenous pressure interfered with gut microbial competition and increased the *Kitasatospora* and *Streptomyces*, which are multi-antibiotic-resistant bacteria and antibiotic producers. The induced multi-antibiotics and antibiotic synergist (clavulanic acid) amplify the antimicrobial effects. Additionally, CTC enhanced microbial synthesis pathways of nutrients and secondary bile acids in the host. (Right) MCE increased *Lactobacillus* to benefit the host in many aspects, such as producing vitamins and generating lactate for anaerobic bacteria to produce butyrate, an anti-inflammatory compound and energy source for the intestine. Some bacteria were competitively inhibited by *Lactobacillus*. Additionally, MCE promoted the synthesis pathways of amino acids, vitamins, and secondary bile acids to provide nutrition for the host

Since the ban on growth-promoting antibiotics in many countries, large-scale chicken farming has faced challenges of prophylaxis and growth promotion. With increasing global consumption of chickens, it has become imperative to develop effective alternatives. Therefore, the well understanding of the growth-promoting mechanism is in great need. Here, we performed the treatment experiment with the dietary supplementation of MCE, and analyzed the changes in chickens as well as in the intestinal microbiome. In general, MCE improved chicken growth performance and modulated intestinal microbiota. Obvious microbial changes were found in the foregut, where nutrients were primarily absorbed, including the increase of beneficial bacteria such as *Lactobacillus*, and the enrichment of biosynthesis pathways of amino acids, vitamins, and secondary bile acids. Moreover, the increased *Lactobacillus* competitively inhibited some pathogens, possibly resulting in the alleviation of host inflammation and immune responses. In addition, our analysis also unveils some of the underlying mechanism of CTC, for comparison with MCE (Fig. 5). Taken together, these findings deepen our understanding of growth promoters in livestock, and provide useful information for the development of safe and effective alternatives to AGPs.

## Methods

### Chickens, diets and experimental design

For the distribution groups (Distribution), five chicken breeds from different commercial providers located in Hunan Changde (DHC), Guangdong Yunfu (DGY), Henan Kaifeng (DHK), Shandong Taian (DST), and Shanxi lvliang (DSL), respectively, in China, were included in this study. Three male and three female individuals were included for four chicken breeds (DHC, DGY, DST, DSL), and only three female individuals were included for one breed that is egg-layer (DHK).

In the treatment experiment groups (growth test), chicken breeds of Arbor Acres broiler (AA) and Local yellow-feather (LY) chickens were studied independently, in two farms in Beijing and Hunan Changsha, respectively. Chickens were randomly divided into 5 groups (12 chickens/repeat, 10 repeats/group) for a 42-day (AA) and 56-day (LY) feeding trail, respectively. The treatments were as follows: (1) BLANK, the basal diet; (2) CTC, the basal diet plus antibiotic (50 mg/kg Citifac®, chlortetracycline 20% *w*/*w* premix); (3) MCE-L, the basal diet plus plant extract (15 mg/kg Sangrovit®, *Macleaya cordata* extract 3.75% *w*/*w* premix); (4) MCE-M, the basal diet plus plant extract (50 mg/kg Sangrovit®, *Macleaya cordata* extract 3.75% *w*/*w* premix); and (5) MCE-H, the basal diet plus plant extract (150 mg/kg Sangrovit®, *Macleaya cordata* extract 3.75% *w*/*w* premix). The basal diets were based on the *Nutrient Requirements of Poultry*: *Ninth Revised Edition*, 1994 (NRC, 1994) and *Feeding Standard of Chicken* (NY/T 33-2004). The chickens had free access to feed and water, and were housed in wired three-level battery cages (100 cm long × 80 cm wide × 40 cm high/cage). The lighting schedule was 20 h light and 4 h dark throughout the experiment. The room temperature was controlled with heaters and gradually reduced from 35 °C on day 1 to 24 °C on day 21 and then kept roughly constant. The chickens were vaccinated using combined Newcastle disease virus (NDV) and infectious bronchitis virus on day 7 through intranasal and intraocular administration, and on day 21 via oral administration. Body weight and feed intake of AA chickens were recorded for each replicate on day 42, while that of LY chickens were recorded for each replicate on day 56.

### Collection of intestinal tissue and digesta samples

To collect intestinal tissue samples of AA chickens (day 21 and day 42), one randomly selected chicken individual from each repeat (ten repeats/group) was slaughtered, and the mid-segment (intestinal tissue) of the ileum was harvested, frozen using liquid nitrogen, and transported to the laboratory in a dry-ice pack, and then kept at − 80 °C until quantification of gene expression.

To collect intestinal digesta samples, randomly chosen chickens from each group (DST, DSL, DGY, DHC, DHK, AA, LY) were slaughtered, and then the duodenum, jejunum, ileum, cecum, and colorectum were immediately removed and dissected. Fresh digesta samples were collected, frozen using liquid nitrogen, and transported to the laboratory in a dry-ice pack, then stored at − 80 °C until DNA extraction. In the treatment experiment groups (growth test), for each intestinal compartment, five digesta samples from five chicken individuals of the same breed (AA and LY chickens), same treatment group (BALNK, CTC, MCE-L, MCE-M, and MCE-H) and same age (7, 14, 28, 42 days), were pooled as one sample for DNA extraction. The five digesta samples from day 1 chickens before growth promoter treatment were also pooled before DNA extraction. For all the other digesta samples from adult chickens, including 135 samples from distribution groups (DST, DSL, DGY, DHC, DHK) and 150 samples from treatment groups (AA chickens, day 42; LY chickens, day 56), each digesta sample from each individual was processed separately.

### RNA isolation and quantitative real-time polymerase chain reaction

Total RNA from the intestinal tissue was extracted by using Trizol reagent (Invitrogen Life Technologies, Carlsbad, CA) according to the manufacturer’s protocol. The concentration and purity of RNA were determined using the NanoDrop 2000 spectrophotometer (Thermo Scientific, Waltham, MA). One microgram of total RNA from each sample was reverse transcribed into cDNA using the PrimeScript RT reagent kit with cDNA eraser (TaKaRa, Dalian, China). The primers used for the reverse transcription were oligo (dT) primer and random hexamers. The one-step real-time PCR was performed with the SYBR® Premix Ex TaqTM (TaKaRa, Dalian, China) using a ABI 7500 fluorescence quantitative PCR machine (Applied Biosystems, Foster City, CA) following the manufacturer’s guidelines. Primers used in this study were listed in Additional file 30: Table S9. Relative mRNA expression levels of each target gene (β-actin, IFN-γ, IL-4, TNF-α, iNOS, NF-κB) were calculated using the 2^−ΔΔCT^ method.

### DNA extraction, library preparation, and sequencing

The bacteria cells were separated from undigested feed particles and recovered through differential centrifugation before cell lysis [70]. A combination of lysis steps was applied. Cells were subjected to five freeze-thaw cycles (alternating between 65 °C and liquid nitrogen for 5 min), followed by repeated beads-beating in ASL buffer (cat. no. 19082; Qiagen Inc.) plus incubation at 95 °C for 15 min. DNA was isolated following a previously reported protocol [71]. Metagenomic DNA paired-end libraries were prepared with an insert size of 350 base pairs (bp) following the manufacture’s protocol (cat. no. E7645L; New England Biolabs). Sequencing was performed on Illumina HiSeq 2500 and HiSeq X10.

### Metagenome assembly and construction of the gene catalog

Raw reads were cleaned to exclude adapter sequences, low-quality sequence, as well as contaminated DNA including host and food genomic DNA. The average error rate of the clean reads is lower than 0.001. The reads that mapped to chicken, human, maize, soybean, wheat, and zebrafish genomes by BWA-MEM were filtered out [72]. Finally, short reads (length < 75-bp) and unpaired reads were also excluded to form a clean reads data.

For each sample, the clean reads were assembled by Megahit (v1.0.6) under pair-end mode respectively [73], then gene prediction was performed on contigs larger than 500-bp by Prodigal (v2.6.3) with parameter “-p meta” [74], and gene models with cds length less than 102-bp were filtered out. As Megahit is a memory efficient assembly software, in theory, it can assemble reads from all the samples together at once, to improve the assembly result for less abundant species. Here, due to the memory limitation of our computer server, all the 495 samples were firstly divided into 5 study groups: distribution groups (135 samples), treatment groups for LY chickens with 56 days (75 samples), treatment groups for LY chickens with 1–42 days (105 samples), treatment groups for AA chickens with 42 days (75 samples), and treatment groups for AA chickens with 1–42 days (105 samples). Then, assembly and gene prediction were performed on these five study groups independently, using the same methods for each sample.

A non-redundant gene catalog was constructed using the gene models predicted from each sample and each group by cd-hit-est (v4.6.6) [75] with parameter “-c 0.95 -n 10 -G 0 -aS 0.9,” which adopts a greedy incremental clustering algorithm and the criteria of identity > 95% and overlap > 90% of the shorter genes. By using the gene models predicted from each sample only, we obtained 6 M non-redundant genes; by adding the additional gene models predicted from each group, finally, we obtained a total of 9 M non-redundant genes.

### Taxonomic and functional assignment of genes

Taxonomic assignments of protein sequences were made on the basis of DIAMOND (v0.8.28.90 diamond blastp --evalue 10 --max-target-seqs 250) alignment against the NCBI-NR database by CARMA3 (carma --classify-blast --type p --database p) [76, 77]. A number of 64,332 genes (0.71%) classified as eukaryota but not fungi were excluded from the non-redundant gene set, and the final chicken gut gene catalog includes 9,037,241 genes.

The functional assignments of protein sequences were made on the basis of DIAMOND alignment against the KEGG protein database (release 79) and eggNOG (v4.5) [78, 79], by taking the best hit with the criteria of *E* value < 1e-5. The annotation of ARG protein sequences were made on the basis of DIAMOND alignment against the Comprehensive Antibiotic Resistance Database (CARD) [80], with the AMR detection models (protein homolog models) provided by the database.

To calculate of relative gene abundance, the clean reads from each sample were aligned against the gene catalog by BWA-MEM with the criteria of alignment length ≥ 50 bp and identity > 95%. Sequenced-based abundance profiling was performed as previously described [81]. Phylum, genus, species, KO, and OG relative abundances were calculated by summing the abundance of the respective genes belonging to each category per sample, based on the taxonomic assignments, KO and OG annotations, respectively. The relative gene abundance profile was also summarized into KEGG and eggNOG functional profiles for the functional analysis. The gene relative abundance profiles and sequences of integrated gene catalog (IGC) of human gut microbiome [16], and the reference gene catalog of the pig gut metagenome [17], were downloaded and analyzed by the same KEGG and eggNOG functional annotation pipeline in our study.

### Microbial composition analysis

For microbial diversity analysis, Shannon index was used. The overall differences in the bacterial community structures were evaluated by non-metric multidimensional scaling (NMDS) based on Bray-Curtis dissimilarity values and performed with “Phyloseq” package in R.

### Co-occurrence network analysis

We calculated the Spearman’s rank correlation coefficient through R package of “ccrepe” between genera, based on the relative abundance profile of genera. Networks were then constructed by using the method implemented in Cytoscape (v3.6) [82].

### Statistical analysis

The significant functional differences between the chicken, human, and pig gut samples were determined by the Wilcoxon rank-sum test, adjusted by the Storey’s methods for multiple tests. Because of the different gene annotation ratios of the chicken, human, and pig gut catalogs, the KEGG functional profiles of the chicken, human, and pig samples were normalized before comparison (genes with no functional annotations were excluded). To avoid the influence of the different intestinal compartments, only when subgroups of the five chicken intestinal compartments all showed the significant difference (*P* < 0.005) compared to the human samples (1267 samples) or pig samples (287 samples), the asterisk was shown in Fig. 1f.

To determine the taxonomic and functional differences between the foregut and hindgut microbial communities, the Wilcoxon rank-sum test (Storey’s methods for multiple tests adjustment) was applied. For the comparison of treatment groups, the Kruskal-Wallis test (Storey’s methods for multiple tests adjustment) was applied, followed by the post-hoc Wilcoxon rank-sum test.

The data of growth performance and qPCR were analyzed with one-way ANOVA and Duncan’s multiple comparison.

## Additional files

Additional file 1: **Table S1.** Background information on the chicken samples. (XLSX 34 kb)

Additional file 2: **Figure S1.** Rarefaction curves of detected genes from the whole set of 495 samples (Total) and from subgroups of each intestinal compartment (99 samples). D (duodenum), J (jejunum), I (ileum), C (cecum), R (colorectum). The gene number of a specific number of samples was calculated after random samplings repeated 100 times with replacement, and the median was plotted. (PDF 211 kb)

Additional file 3: **Table S2.** Comparison of gut microbial gene catalogs of human, pig and chicken. (XLSX 38 kb)

Additional file 4: **Table S3.** Significant differences between the foregut and hindgut of chickens at the phylum and genus levels. (XLSX 40 kb)

Additional file 5: **Figure S2.** Functional annotation of gut microbial genes based on KEGG orthologous groups (KOs) and eggNOG orthologous groups (OGs). (**a**) Comparison of the total gene numbers and the functionally annotated gene numbers of the chicken, human and pig catalogs. (**b**) Comparison of the number of KOs and OGs presented in the chicken, human and pig catalogs. (PDF 165 kb)

Additional file 6: **Figure S3.** Venn diagram of eggNOG orthologous groups (OGs) presented in and shared by the chicken, human and pig catalogs. (PDF 103 kb)

Additional file 7: **Figure S4.** The NMDS plot of the chicken, human and pig gut samples based on Bray-Curtis dissimilarities at KO level. (PDF 900 kb)

Additional file 8: **Figure S5.** Microbial community compositions of the duodenum, jejunum, ileum, cecum, and colorectum across 495 chicken gut samples. (**a**) Phylum-level compositions. (**b**) Genus-level compositions. “Others” refers to all the other phyla or genera in the samples (unclassified not included). (PDF 429 kb)

Additional file 9: **Figure S6.** The NMDS plot of foregut samples in five Distribution groups (DGY, DHC, DHK, DSL, DST) based on Bray-Curtis dissimilarities at the species level. (PDF 887 kb)

Additional file 10: **Figure S7.** Microbial diversity (Shannon index) of samples in five Distribution groups (DGY, DHC, DHK, DSL, DST) at gene, genus, OG and KO levels. Box plots show median ± interquartile range (IQR) and 1.5 IQR ranges (whiskers), with outliers denoted by dots. (PDF 866 kb)

Additional file 11: **Figure S8.** Average relative abundance of phylum *Actinobacteria* of foregut samples in five Distribution groups (DGY, DHC, DHK, DSL, DST). (PDF 744 kb)

Additional file 12: **Table S4.** The number of shared genes of samples in each intestinal compartment of chickens. (XLSX 53 kb)

Additional file 13: **Figure S9**. Comparison of KEGG functional profiles of five intestinal compartments. The average relative abundance of samples in each KEGG functional category was plotted. (PDF 760 kb)

Additional file 14: **Figure S10.** Microbial gene functional differences between foregut and hindgut though eggNOG annotation (Wilcoxon rank-sum test, Storey’s methods for multiple tests adjustment). (PDF 812 kb)

Additional file 15: **Figure S11.** Comparison of eggNOG functional profiles of five intestinal compartments. The average relative abundance of samples in each eggNOG functional categories was plotted. (PDF 773 kb)

Additional file 16: **Figure S12.** The NMDS plot of microbial communities in hindgut at different ages. The analysis was based on Bray-Curtis dissimilarities at species level and samples were grouped according to the ages. (PDF 194 kb)

Additional file 17: **Figure S13.** Microbial diversity (Shannon index) at gene, genus, OG and KO levels of hindgut samples at different ages. Box plots show median ± interquartile range (IQR) and 1.5 IQR ranges (whiskers), with outliers denoted by dots. (PDF 201 kb)

Additional file 18: **Table S5.** The relative abundances of major phyla at different ages (average relative abundance > 0.01%). (XLSX 40 kb)

Additional file 19: **Table S6.** The relative abundances of major genera at different ages (average relative abundance > 0.01%). (XLSX 44 kb)

Additional file 20: **Figure S14.** The relative abundance of major genera in the foregut and hindgut at different ages. The average relative abundance of samples was plotted. (PDF 552 kb)

Additional file 21: **Figure S15.** The differences in KEGG functional pathways of the microbiome in the hindgut at different ages. (PDF 172 kb)

Additional file 22: **Figure S16.** The NMDS plot of microbial communities in BLANK and MCE groups (left); and in BLANK and CTC (right). The analysis was based on Bray-Curtis dissimilarities at the species level. (PDF 877 kb)

Additional file 23: **Figure S17.** The influences of CTC and MCE on microbial diversity (Shannon index) at gene, genus, OG and KO levels (**a**) in the foregut and (**b**) in the hindgut. Box plots show median ± interquartile range (IQR) and 1.5 IQR ranges (whiskers), with outliers denoted by dots. Asterisks denote the significant changes (Wilcoxon rank-sum test, *P* < 0.05) between BLANK and the growth promoter treated group. (PDF 859 kb)

Additional file 24: **Figure S18.** (**a**) The relative abundances of genera increased by MCE or CTC in the foregut. *Kitasatospora* and *Streptomyces* were significantly (*P* < 0.05) increased by CTC. (**b**) The relative abundances of antibiotic biosynthesis pathways were significantly (*P* < 0.05) increased by CTC. (**c**) The relative abundances of antibiotic resistance genes (ARGs). ARGs were increased (*P* < 0.1) by CTC. Box plots show median ± interquartile range (IQR) and 1.5 IQR ranges (whiskers), outliers denoted by dots. (PDF 957 kb)

Additional file 25: **Figure S19.** The relative abundances of four major classes of ARGs changed by MCE or CTC in the foregut. Box plots show median ± interquartile range (IQR) and 1.5 IQR ranges (whiskers), outliers denoted by dots. (PDF 823 kb)

Additional file 26: **Figure S20.** The relative abundances of core genera significantly changed by CTC or MCE in the foregut. Box plots show median ± interquartile range (IQR) and 1.5 IQR ranges (whiskers), outliers denoted by dots. Asterisks denote the significant changes (Wilcoxon rank-sum test, *P* <  0.05) between BLANK and the growth promoter treated groups. (PDF 963 kb)

Additional file 27: **Figure S21.** The influences of CTC and MCE on the hindgut microbial functions. The heatmap of KEGG metabolic pathways in hindgut (12 samples for each group, including 6 samples from AA chickens and 6 samples from LY chickens). The relative abundance of each pathway was colored according to its row z-score ((value – row mean)/row standard deviation). Black and white rectangles at the right side of the heatmap represent the significant decrease (*P* < 0.05), and no significant change (*P* > 0.05) compared to BLANK, respectively. The Kruskal-Wallis test (Storey’s methods for adjustment) was used followed by a post-hoc Wilcoxon rank-sum test. (PDF 256 kb)

Additional file 28: **Table S7.** KEGG pathways significantly changed in the foregut after CTC and MCE treatment. (XLSX 13 kb)

Additional file 29: **Table S8.** The relative mRNA levels of cytokines in the ileum of Arbor Acres broiler chickens on days 21 and 42. (XLSX 11 kb)

Additional file 30: **Table S9.** Primers used for quantitative real-time PCR. (XLSX 28 kb)
