# Supplementary figures and images for: The chicken gut metagenome and the modulatory effects of plant-derived benzylisoquinoline alkaloids
Source: Microbiome. 2018 Nov 27;6:211. doi: 10.1186/s40168-018-0590-5 (PMC6260706; doi:10.1186/s40168-018-0590-5)

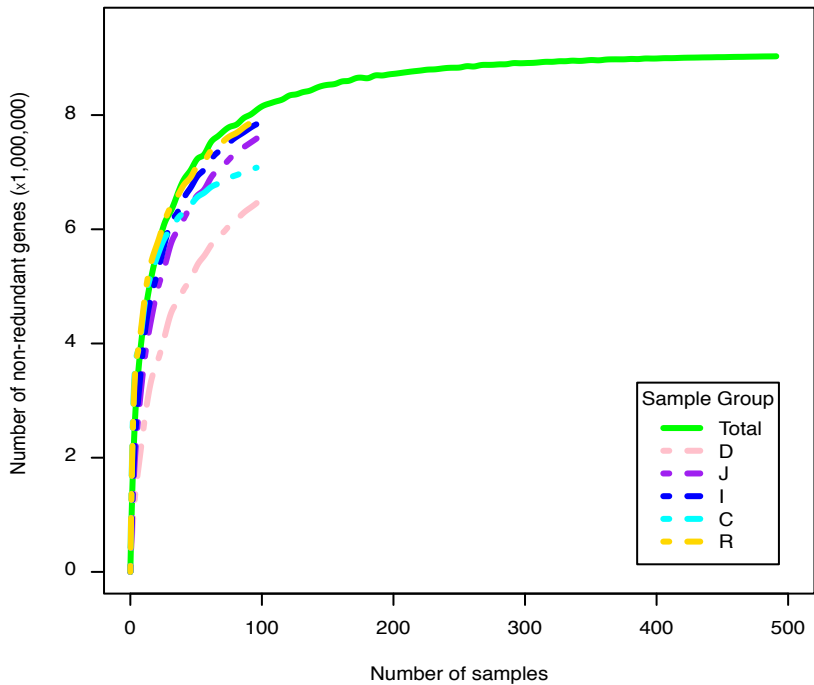

Supplement: Supplementary file 2 — Figure S1. Rarefaction curves of detected genes from the whole set of 495 samples (Total) and from subgroups of each intestinal compartment (99 samples). D (duodenum), J (jejunum), I (ileum), C (cecum), R (colorectum). The gene number of a specific number of samples was calculated after random samplings repeated 100 times with replacement, and the median was plotted. (PDF 211 kb) [file 40168_2018_590_MOESM2_ESM.pdf]

**a**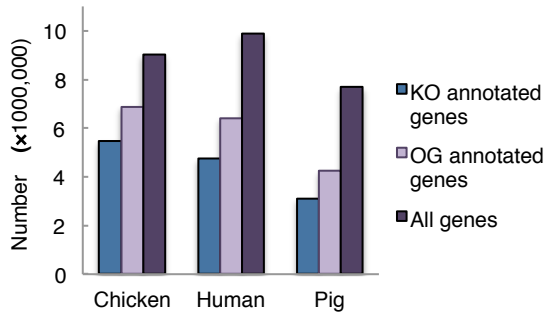**b**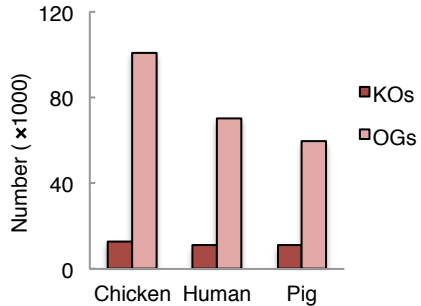

Supplement: Supplementary file 5 — Figure S2. Functional annotation of gut microbial genes based on KEGG orthologous groups (KOs) and eggNOG orthologous groups (OGs). (a) Comparison of the total gene numbers and the functionally annotated gene numbers of the chicken, human and pig catalogs. (b) Comparison of the number of KOs and OGs presented in the chicken, human and pig catalogs. (PDF 165 kb) [file 40168_2018_590_MOESM5_ESM.pdf]

# OG

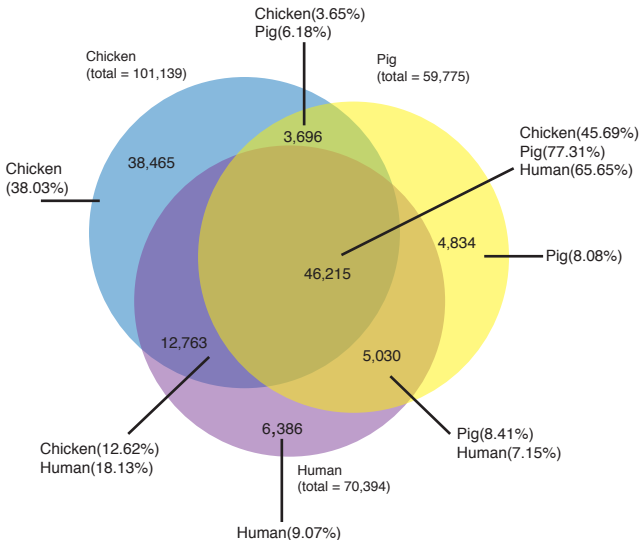

Supplement: Supplementary file 6 — Figure S3. Venn diagram of eggNOG orthologous groups (OGs) presented in and shared by the chicken, human and pig catalogs. (PDF 103 kb) [file 40168_2018_590_MOESM6_ESM.pdf]

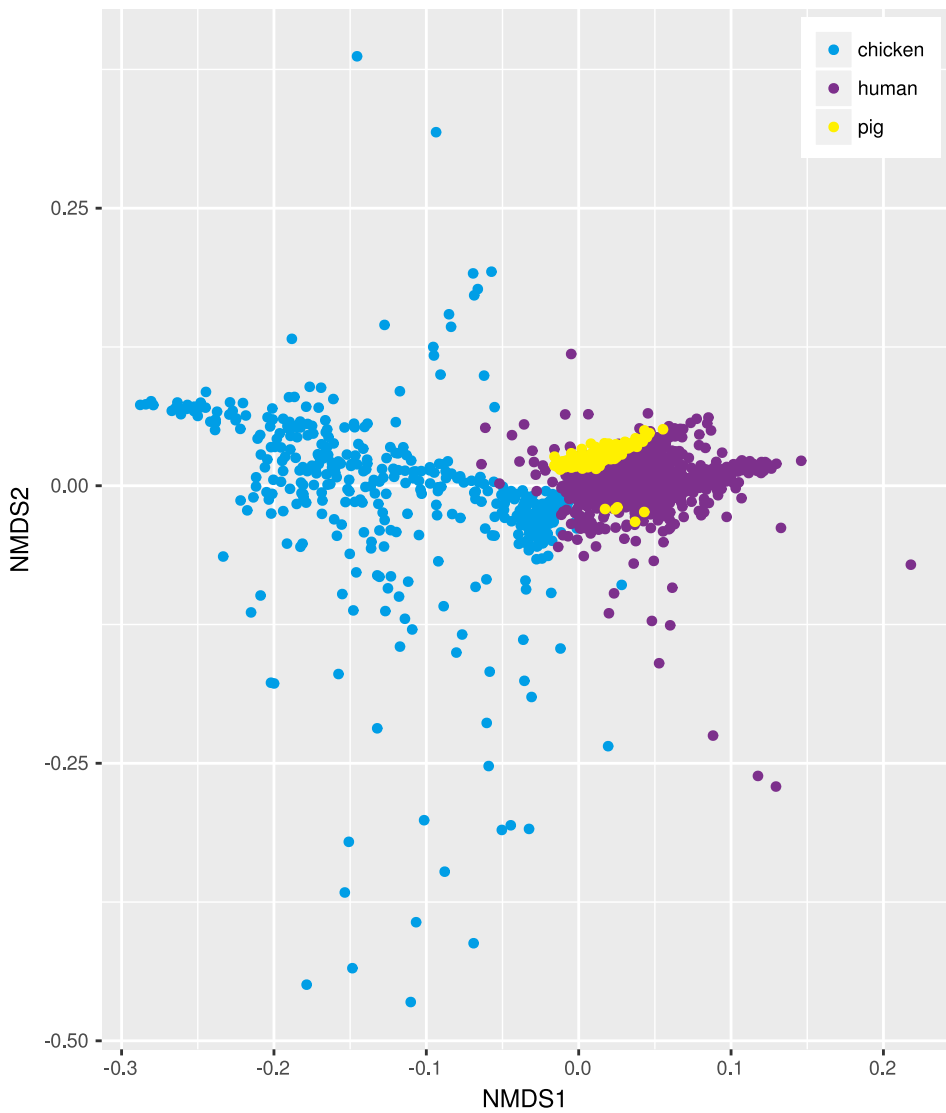

Supplement: Supplementary file 7 — Figure S4. The NMDS plot of the chicken, human and pig gut samples based on Bray-Curtis dissimilarities at KO level. (PDF 900 kb) [file 40168_2018_590_MOESM7_ESM.pdf]

a

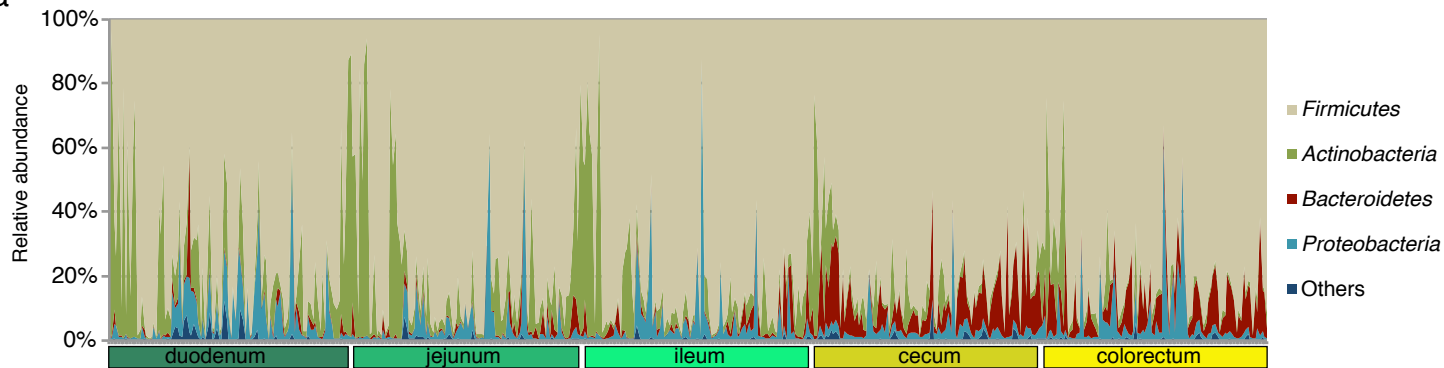

b

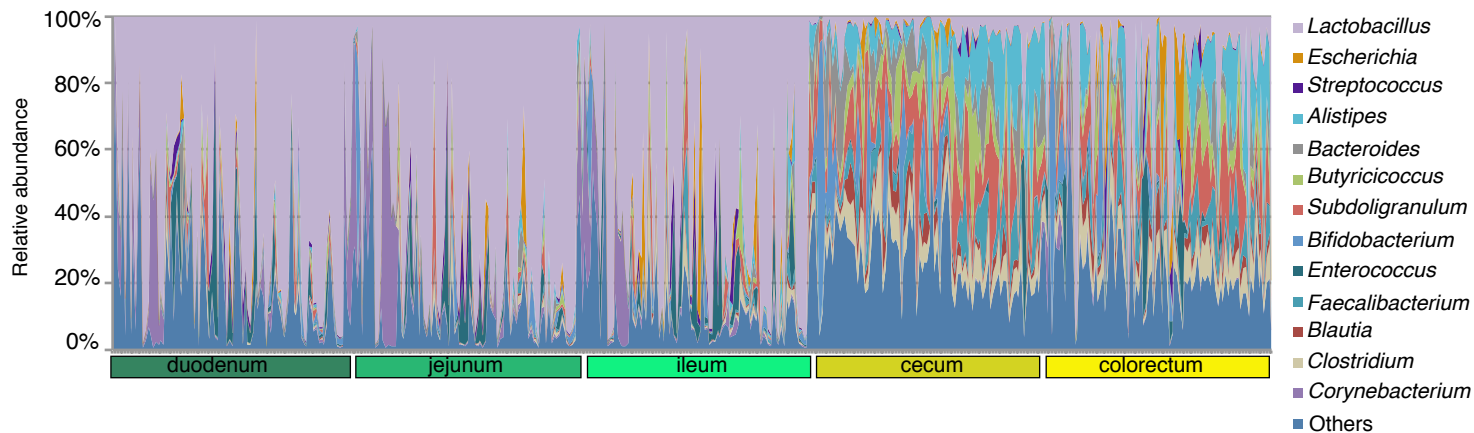

Supplement: Supplementary file 8 — Figure S5. Microbial community compositions of the duodenum, jejunum, ileum, cecum, and colorectum across 495 chicken gut samples. (a) Phylum-level compositions. (b) Genus-level compositions. “Others” refers to all the other phyla or genera in the samples (unclassified not included). (PDF 429 kb) [file 40168_2018_590_MOESM8_ESM.pdf]

NMDS2

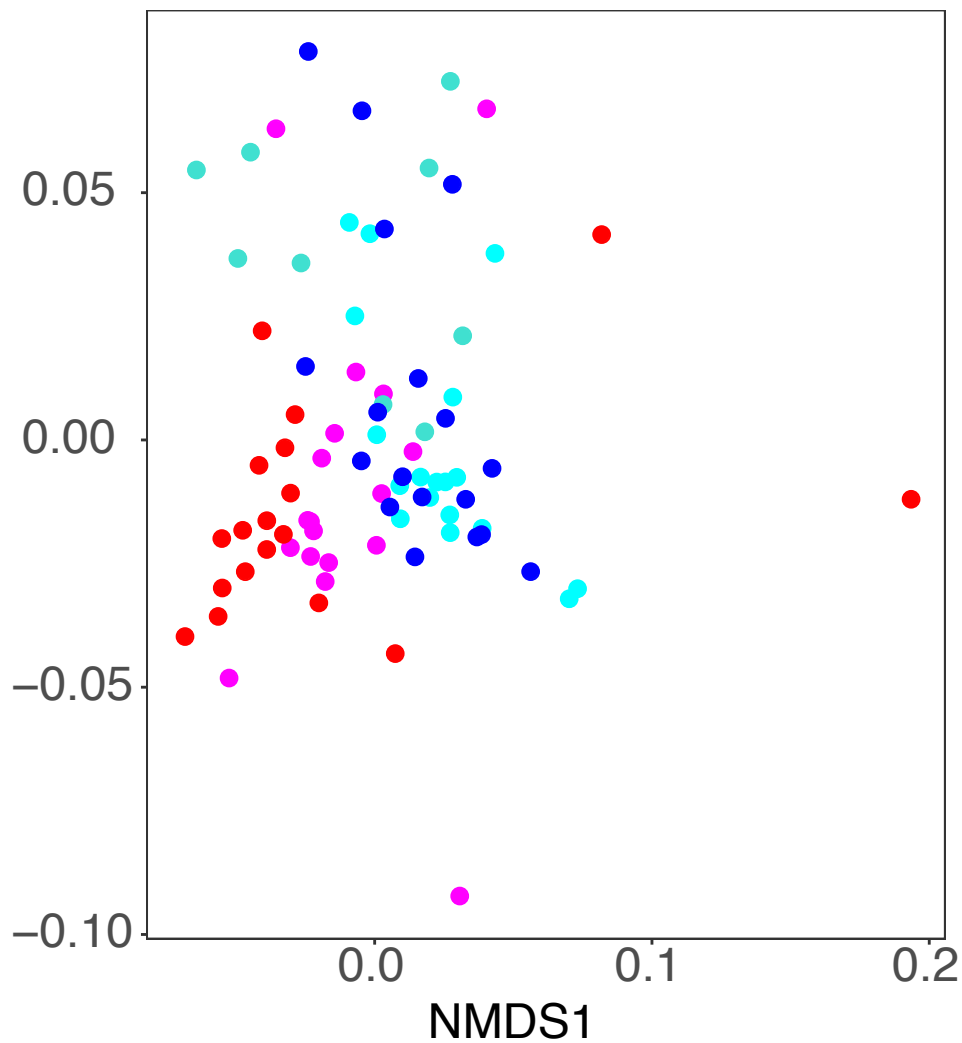

Regions

DGY

DHC

DHK

DSL

DST

Supplement: Supplementary file 9 — Figure S6. The NMDS plot of foregut samples in five Distribution groups (DGY, DHC, DHK, DSL, DST) based on Bray-Curtis dissimilarities at the species level. (PDF 887 kb) [file 40168_2018_590_MOESM9_ESM.pdf]

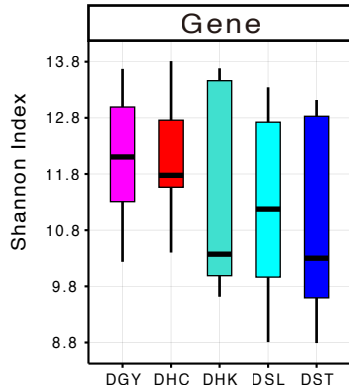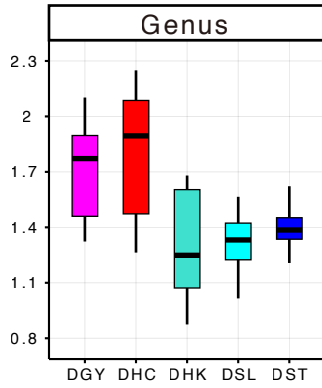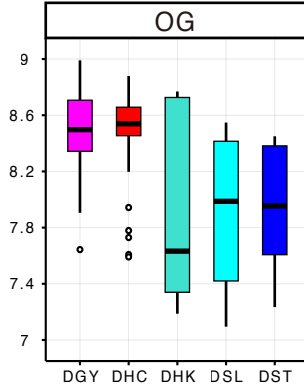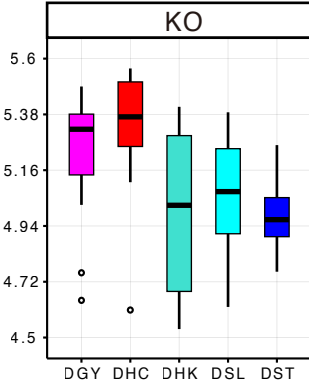

Supplement: Supplementary file 10 — Figure S7. Microbial diversity (Shannon index) of samples in five Distribution groups (DGY, DHC, DHK, DSL, DST) at gene, genus, OG and KO levels. Box plots show median ± interquartile range (IQR) and 1.5 IQR ranges (whiskers), with outliers denoted by dots. (PDF 866 kb) [file 40168_2018_590_MOESM10_ESM.pdf]

phylum  
**Actinobacteria**

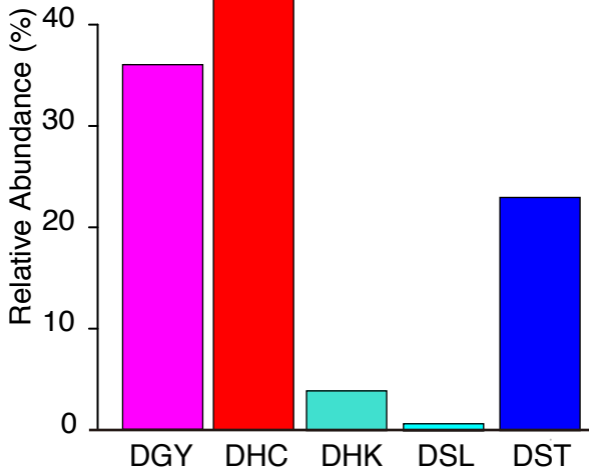

Supplement: Supplementary file 11 — Figure S8. Average relative abundance of phylum Actinobacteria of foregut samples in five Distribution groups (DGY, DHC, DHK, DSL, DST). (PDF 744 kb) [file 40168_2018_590_MOESM11_ESM.pdf]

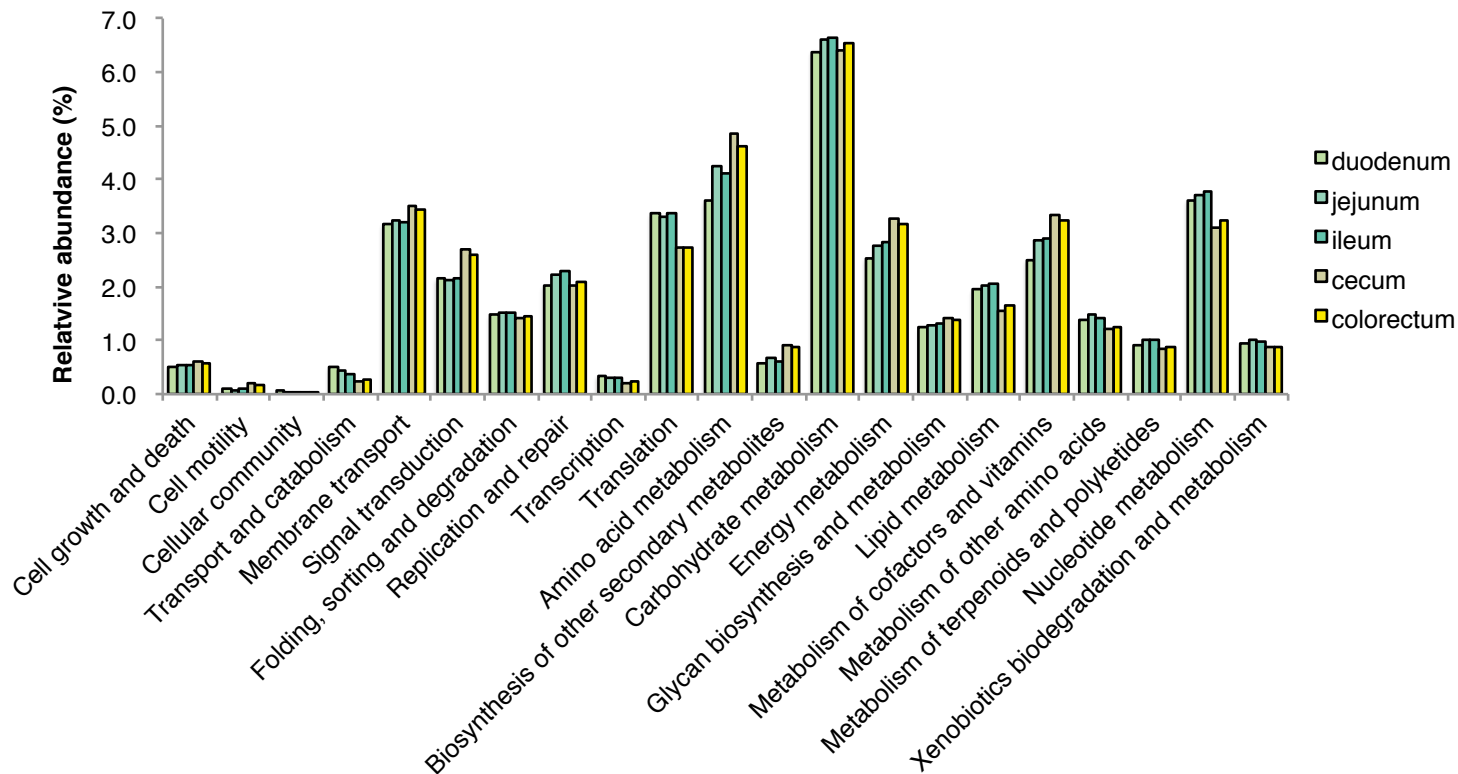

Supplement: Supplementary file 13 — Figure S9. Comparison of KEGG functional profiles of five intestinal compartments. The average relative abundance of samples in each KEGG functional category was plotted. (PDF 760 kb) [file 40168_2018_590_MOESM13_ESM.pdf]

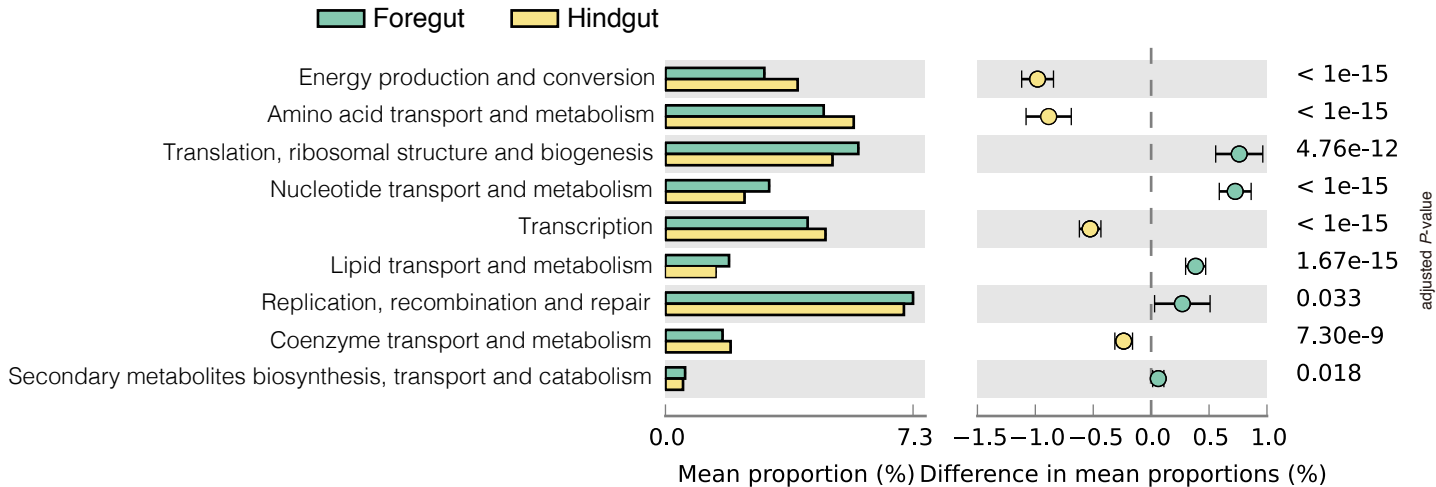

Supplement: Supplementary file 14 — Figure S10. Microbial gene functional differences between foregut and hindgut though eggNOG annotation (Wilcoxon rank-sum test, Storey’s methods for multiple tests adjustment). (PDF 812 kb) [file 40168_2018_590_MOESM14_ESM.pdf]

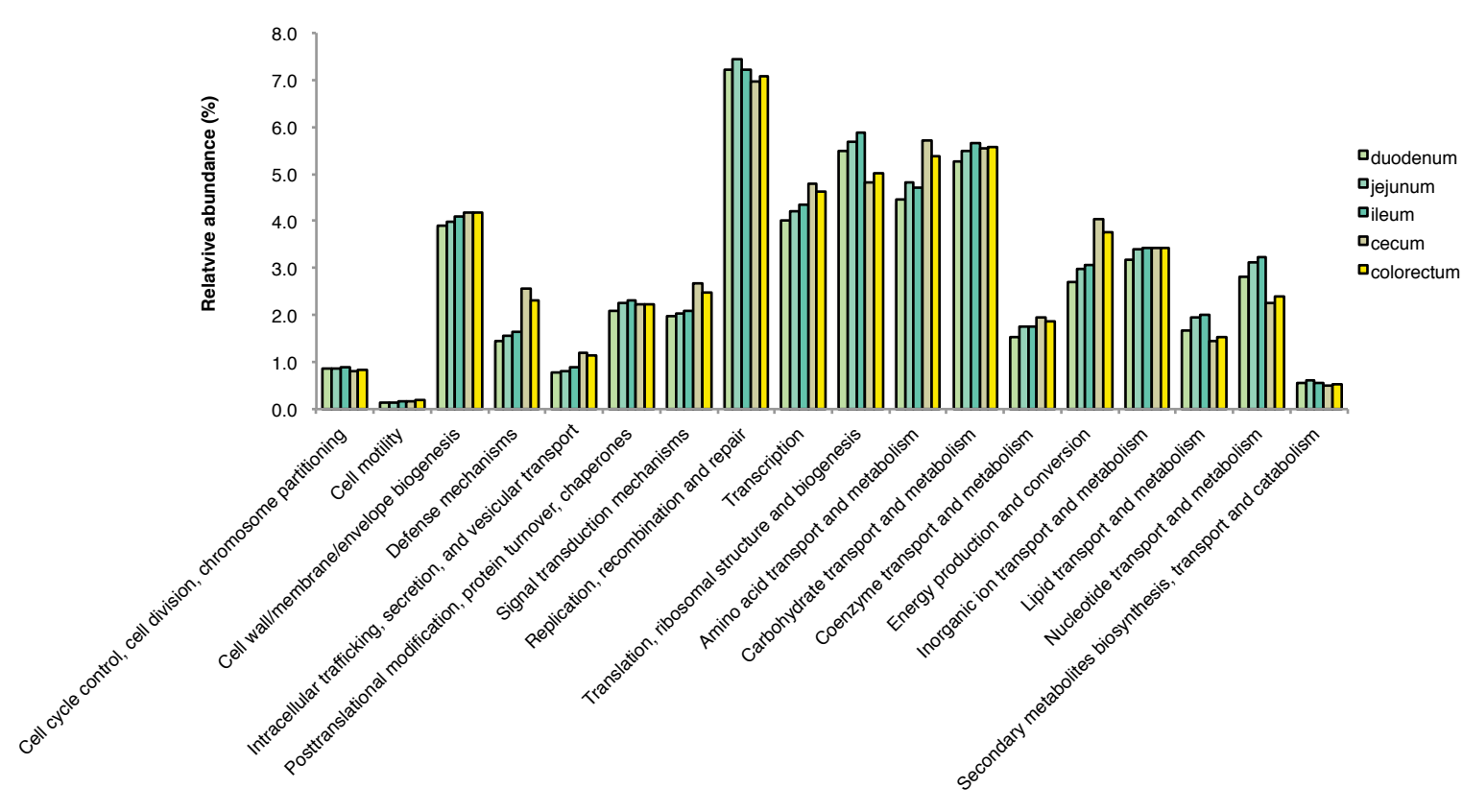

Supplement: Supplementary file 15 — Figure S11. Comparison of eggNOG functional profiles of five intestinal compartments. The average relative abundance of samples in each eggNOG functional categories was plotted. (PDF 773 kb) [file 40168_2018_590_MOESM15_ESM.pdf]

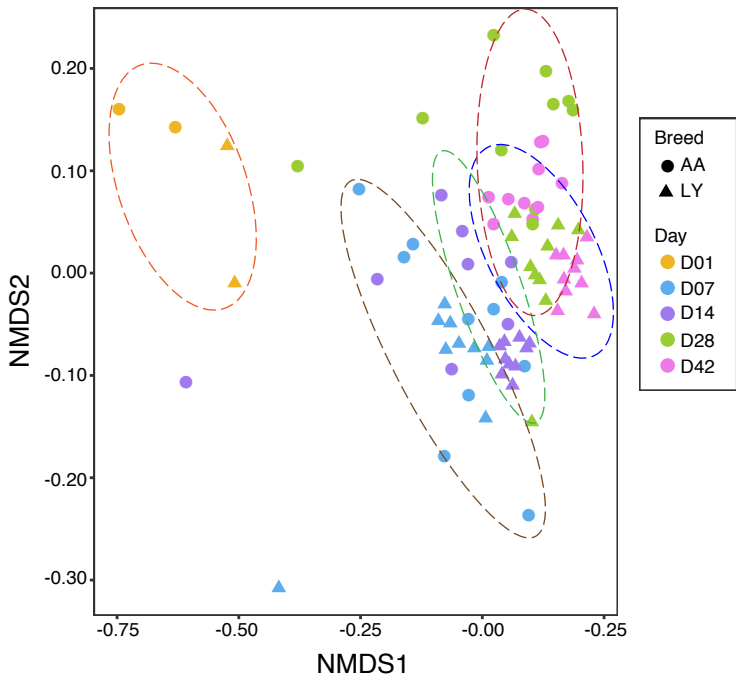

Supplement: Supplementary file 16 — Figure S12. The NMDS plot of microbial communities in hindgut at different ages. The analysis was based on Bray-Curtis dissimilarities at species level and samples were grouped according to the ages. (PDF 194 kb) [file 40168_2018_590_MOESM16_ESM.pdf]

Shannon index

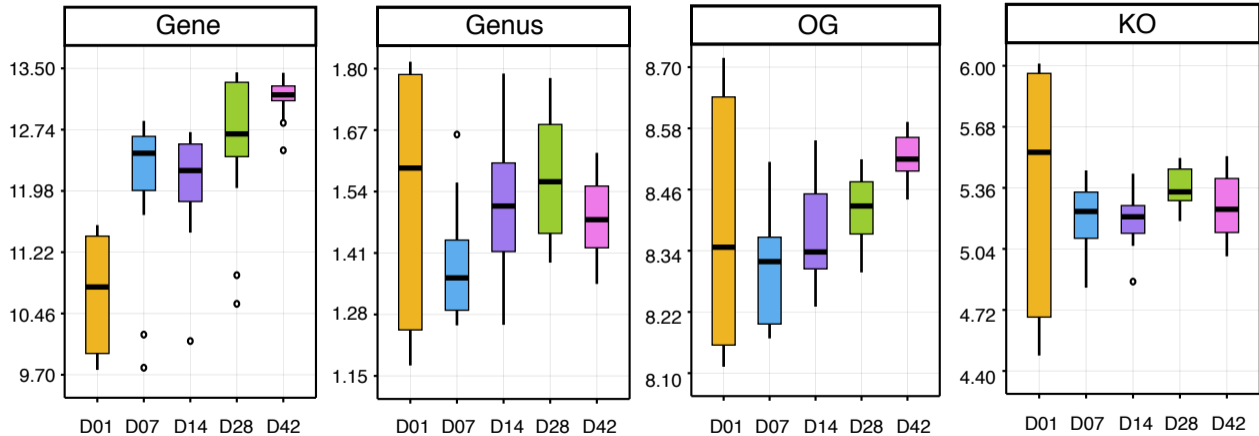

Supplement: Supplementary file 17 — Figure S13. Microbial diversity (Shannon index) at gene, genus, OG and KO levels of hindgut samples at different ages. Box plots show median ± interquartile range (IQR) and 1.5 IQR ranges (whiskers), with outliers denoted by dots. (PDF 201 kb) [file 40168_2018_590_MOESM17_ESM.pdf]

## Genera in foregut

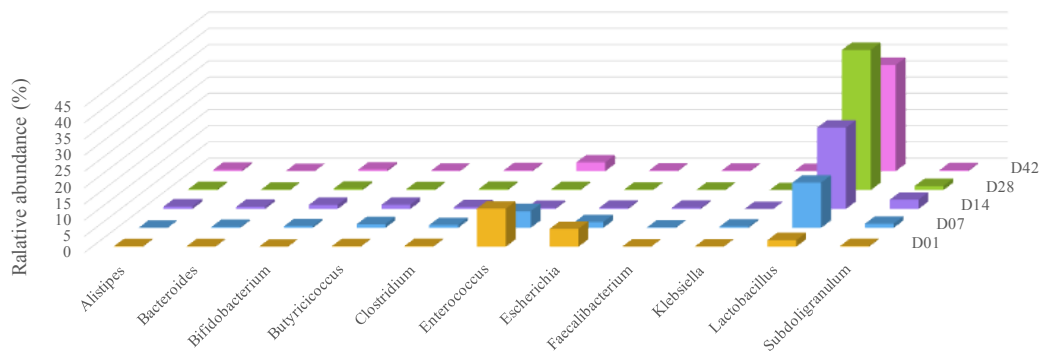

## Genera in hindgut

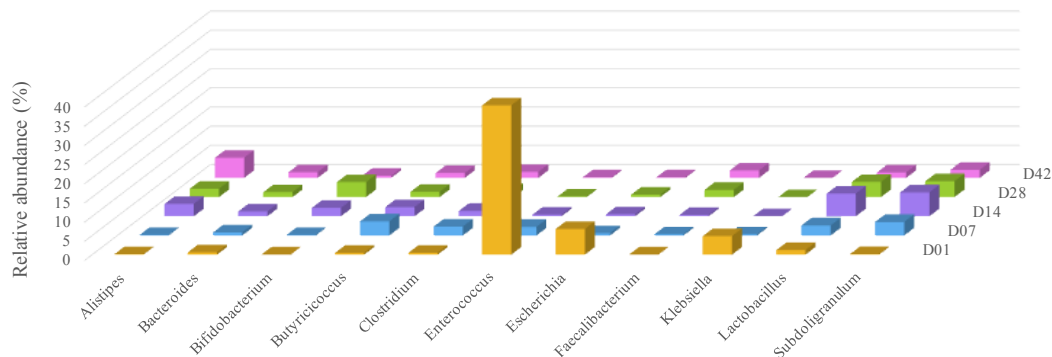

Supplement: Supplementary file 20 — Figure S14. The relative abundance of major genera in the foregut and hindgut at different ages. The average relative abundance of samples was plotted. (PDF 552 kb) [file 40168_2018_590_MOESM20_ESM.pdf]

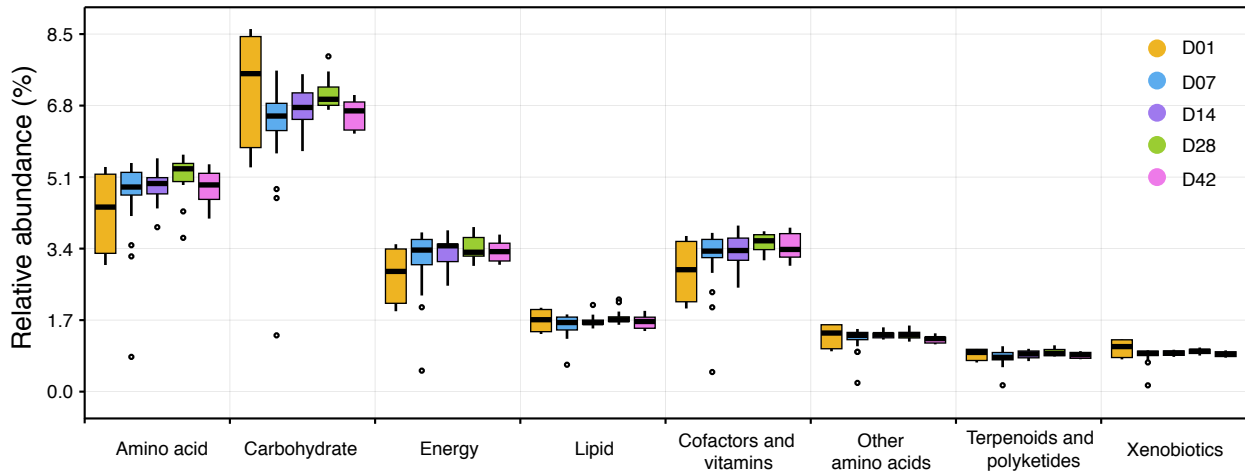

Supplement: Supplementary file 21 — Figure S15. The differences in KEGG functional pathways of the microbiome in the hindgut at different ages. (PDF 172 kb) [file 40168_2018_590_MOESM21_ESM.pdf]

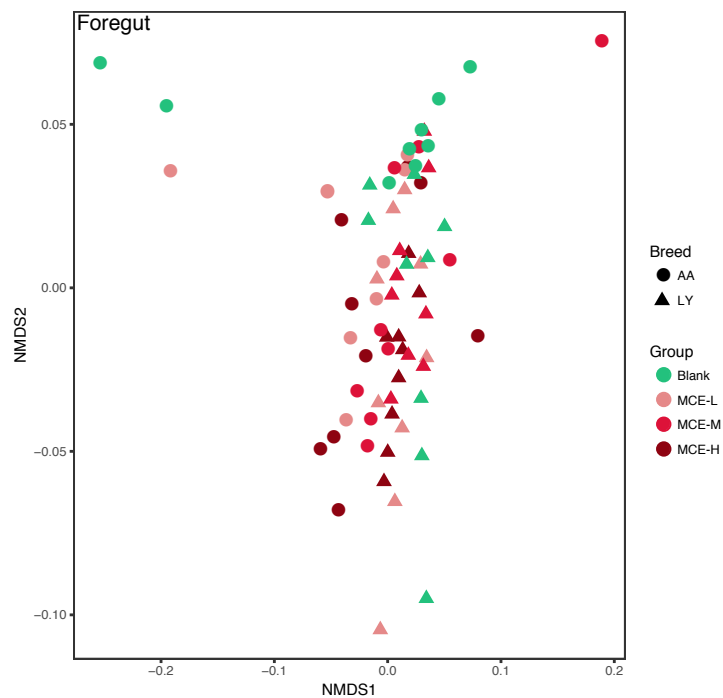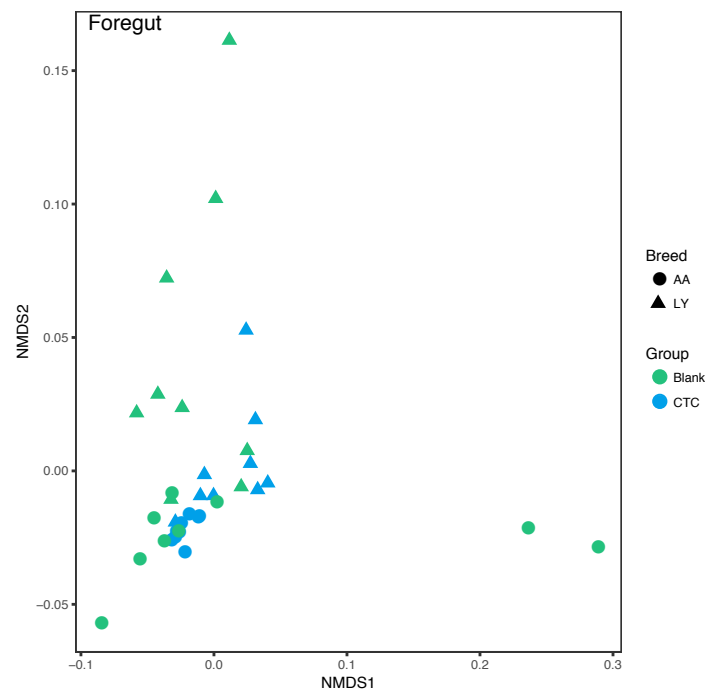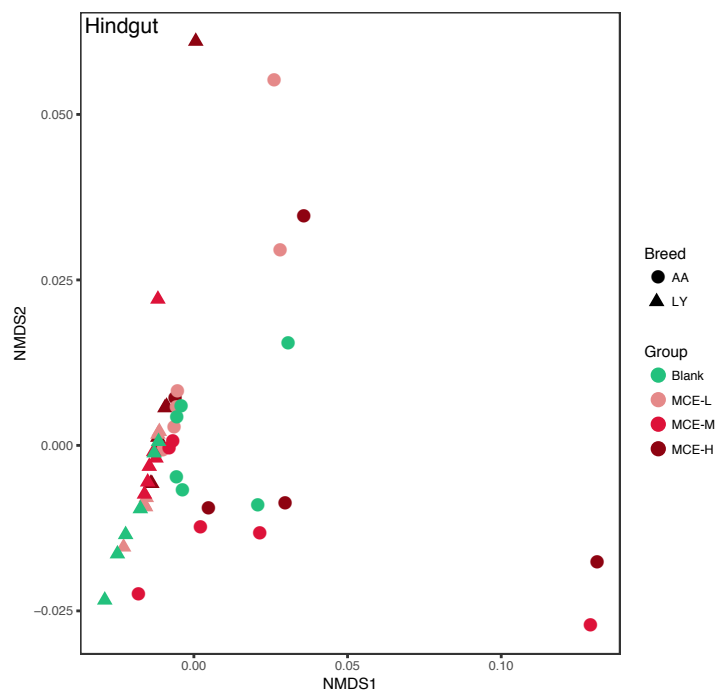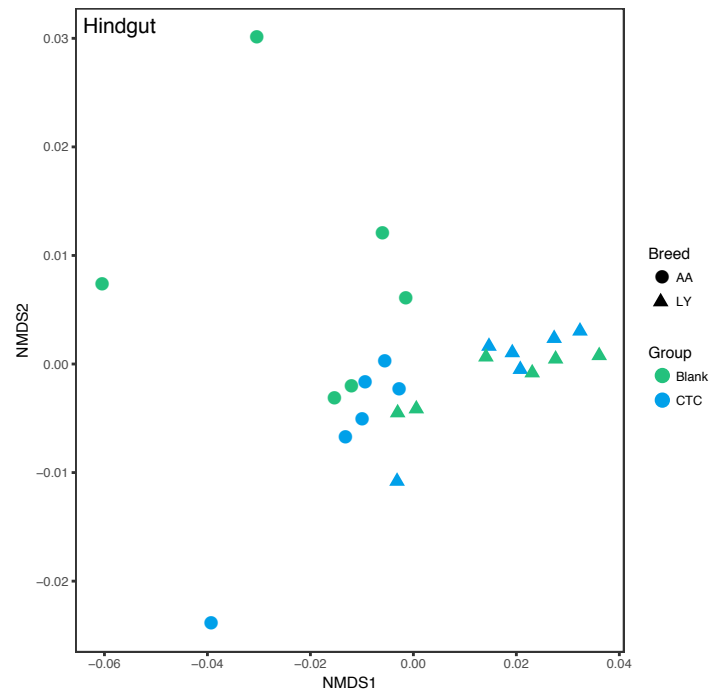

Supplement: Supplementary file 22 — Figure S16. The NMDS plot of microbial communities in BLANK and MCE groups (left); and in BLANK and CTC (right). The analysis was based on Bray-Curtis dissimilarities at the species level. (PDF 877 kb) [file 40168_2018_590_MOESM22_ESM.pdf]

a

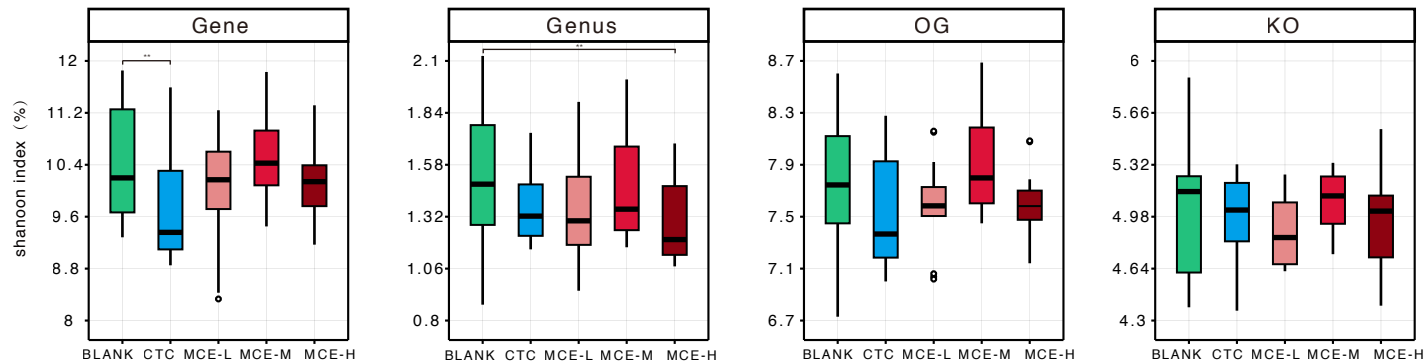

b

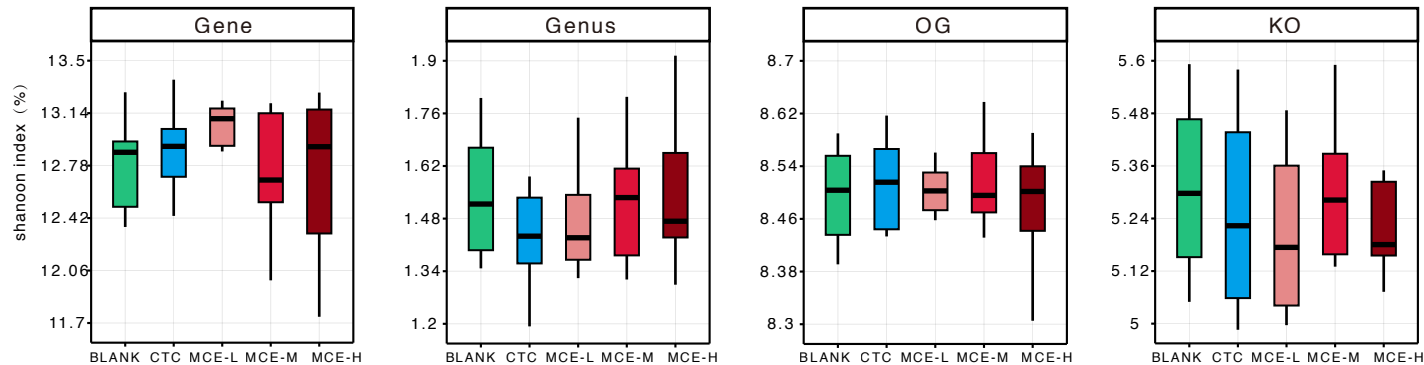

Supplement: Supplementary file 23 — Figure S17. The influences of CTC and MCE on microbial diversity (Shannon index) at gene, genus, OG and KO levels (a) in the foregut and (b) in the hindgut. Box plots show median ± interquartile range (IQR) and 1.5 IQR ranges (whiskers), with outliers denoted by dots. Asterisks denote the significant changes (Wilcoxon rank-sum test, P < 0.05) between BLANK and the growth promoter treated group. (PDF 859 kb) [file 40168_2018_590_MOESM23_ESM.pdf]

a

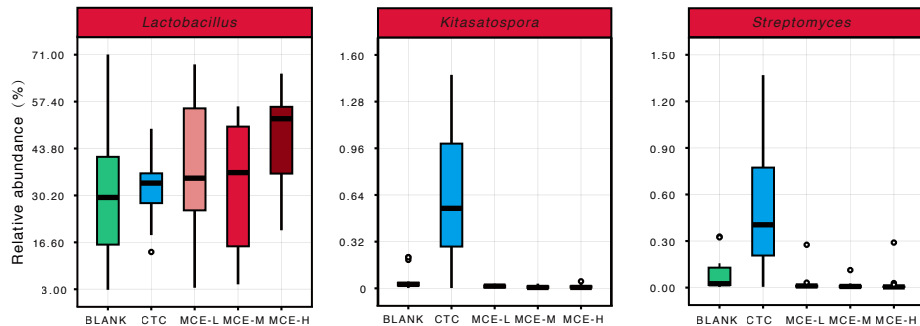

b

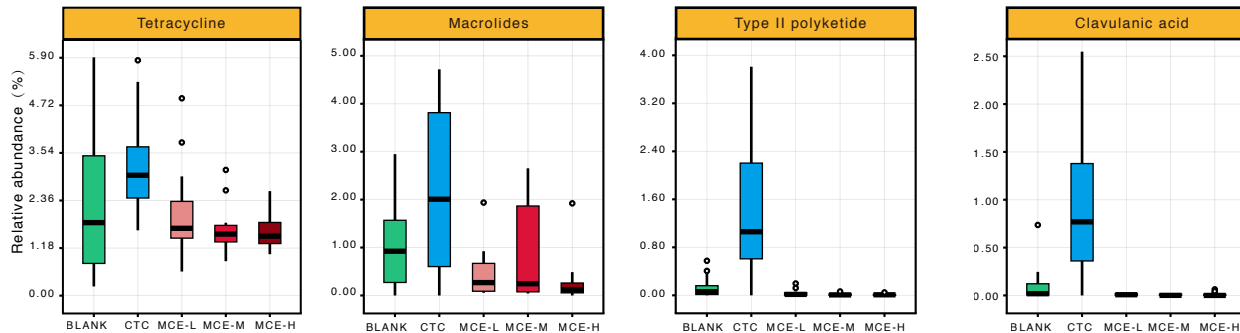

c

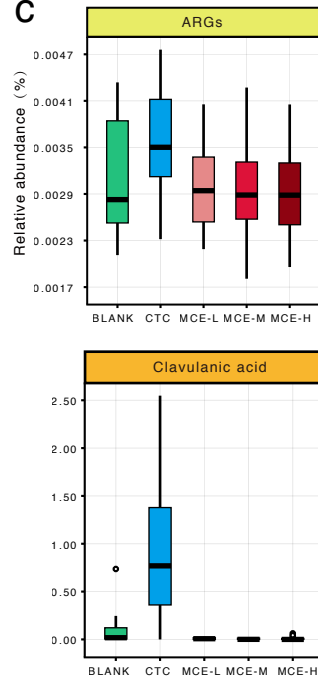

Supplement: Supplementary file 24 — Figure S18. (a) The relative abundances of genera increased by MCE or CTC in the foregut. Kitasatospora and Streptomyces were significantly (P < 0.05) increased by CTC. (b) The relative abundances of antibiotic biosynthesis pathways were significantly (P < 0.05) increased by CTC. (c) The relative abundances of antibiotic resistance genes (ARGs). ARGs were increased (P < 0.1) by CTC. Box plots show median ± interquartile range (IQR) and 1.5 IQR ranges (whiskers), outliers denoted by dots. (PDF 957 kb) [file 40168_2018_590_MOESM24_ESM.pdf]

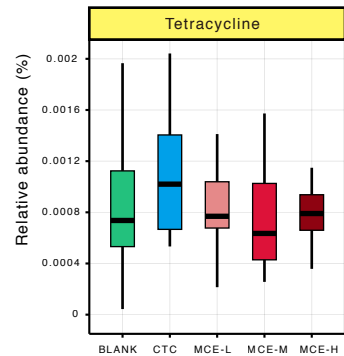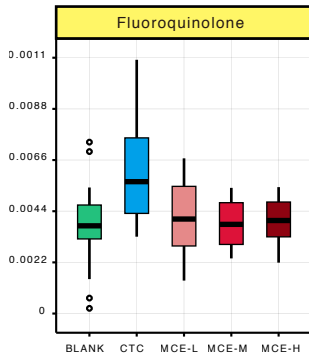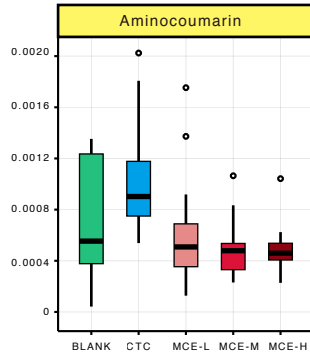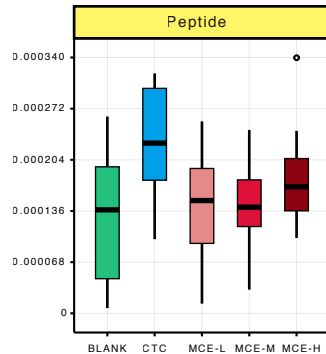

Supplement: Supplementary file 25 — Figure S19. The relative abundances of four major classes of ARGs changed by MCE or CTC in the foregut. Box plots show median ± interquartile range (IQR) and 1.5 IQR ranges (whiskers), outliers denoted by dots. (PDF 823 kb) [file 40168_2018_590_MOESM25_ESM.pdf]

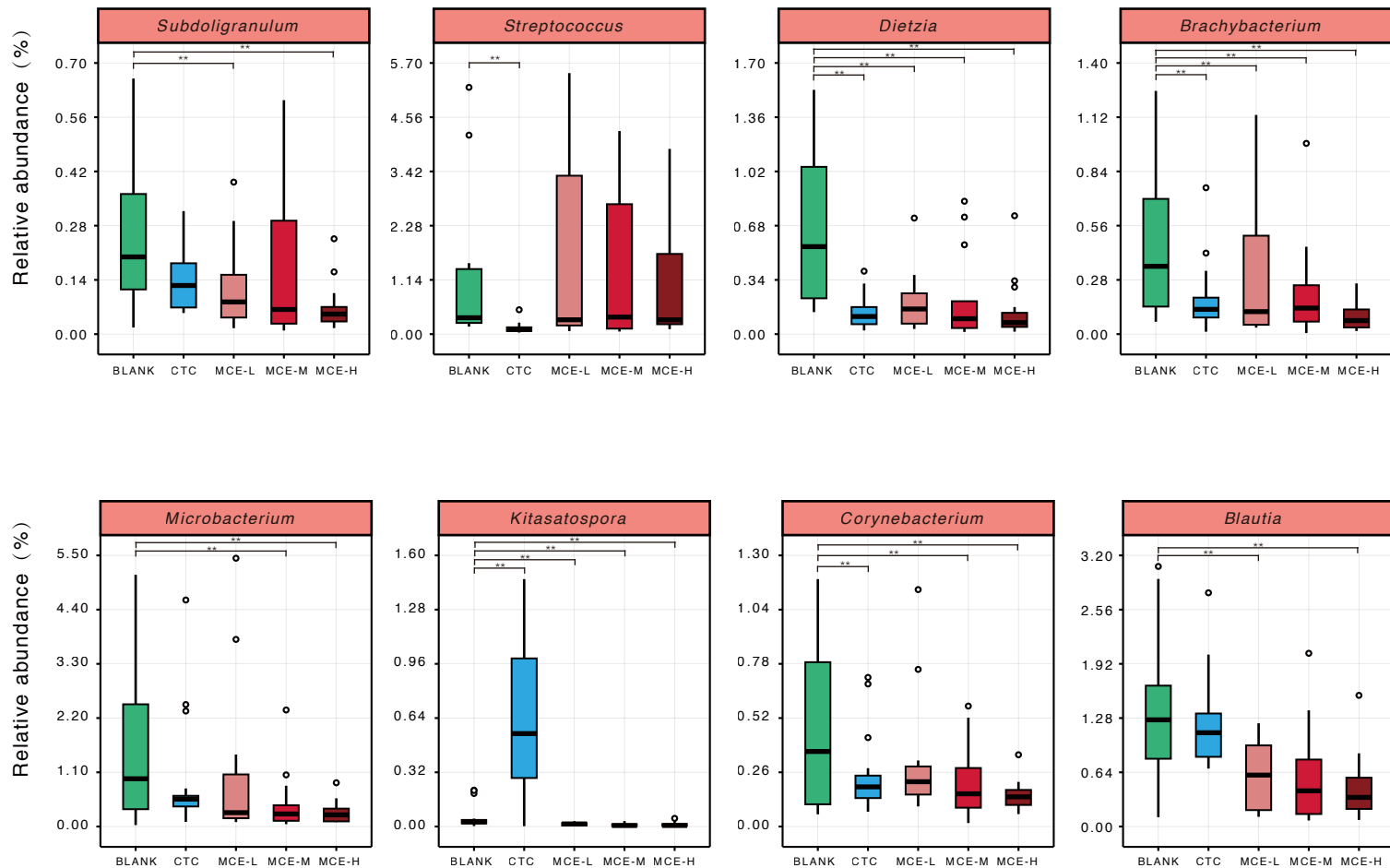

Supplement: Supplementary file 26 — Figure S20. The relative abundances of core genera significantly changed by CTC or MCE in the foregut. Box plots show median ± interquartile range (IQR) and 1.5 IQR ranges (whiskers), outliers denoted by dots. Asterisks denote the significant changes (Wilcoxon rank-sum test, P < 0.05) between BLANK and the growth promoter treated groups. (PDF 963 kb) [file 40168_2018_590_MOESM26_ESM.pdf]

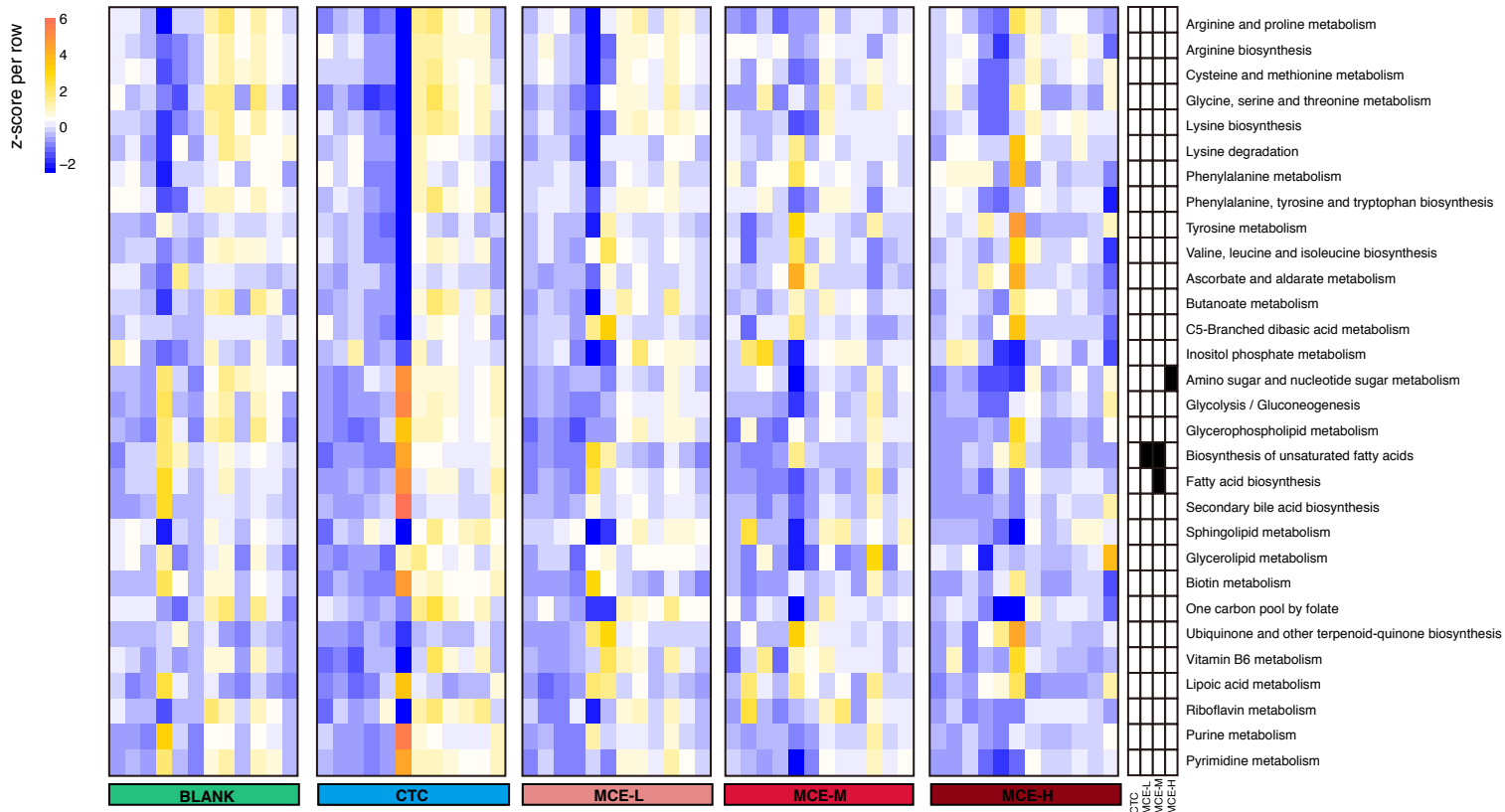

Supplement: Supplementary file 27 — Figure S21. The influences of CTC and MCE on the hindgut microbial functions. The heatmap of KEGG metabolic pathways in hindgut (12 samples for each group, including 6 samples from AA chickens and 6 samples from LY chickens). The relative abundance of each pathway was colored according to its row z-score ((value – row mean)/row standard deviation). Black and white rectangles at the right side of the heatmap represent the significant decrease (P < 0.05), and no significant change (P > 0.05) compared to BLANK, respectively. The Kruskal-Wallis test (Storey’s methods for adjustment) was used followed by a post-hoc Wilcoxon rank-sum test. (PDF 256 kb) [file 40168_2018_590_MOESM27_ESM.pdf]
